# HLH as an additional warning sign of inborn errors of immunity beyond familial-HLH in children: a systematic review

**DOI:** 10.3389/fimmu.2024.1282804

**Published:** 2024-02-13

**Authors:** Silvia Ricci, Walter Maria Sarli, Lorenzo Lodi, Clementina Canessa, Francesca Lippi, Donata Dini, Marta Ferrari, Laura Pisano, Elena Sieni, Giuseppe Indolfi, Massimo Resti, Chiara Azzari

**Affiliations:** ^1^ Department of Health Sciences, University of Florence, Florence, Italy; ^2^ Immunology Division, Section of Pediatrics, Meyer Children’s Hospital IRCCS, Florence, Italy; ^3^ Department of Pediatrics, Meyer Children’s Hospital IRCCS, Florence, Italy; ^4^ Pediatric Hematology-Oncology Department, Meyer Children’s Hospital IRCCS, Florence, Italy; ^5^ Department Neurofarba, University of Florence, Florence, Italy

**Keywords:** hemophagocytic lymphohistiocytosis, inborn errors of immunity, macrophage activation syndrome, immune deficiency, familial hemophagocytic lymphohistiocytosis, hemophagocytic syndrome

## Abstract

**Background:**

Hemophagocytic Lymphohistiocytosis (HLH) is a rare and life-threatening condition characterized by a severe impairment of the immune homeostasis. While Familial-HLH (FHL) is a known cause, the involvement of other Inborn Errors of Immunity (IEI) in pediatric-HLH remains understudied.

**Objective:**

This systematic review aimed to assess the clinical features, triggers, laboratory data, treatment, and outcomes of pediatric HLH patients with IEI other than FHL (IEInotFHL), emphasizing the importance of accurate identification and management.

**Methods:**

A systematic search for studies meeting inclusion criteria was conducted in PubMed, EMBASE, MEDLINE, and Cochrane Central. Quality assessment was performed through JBI criteria.

**Results:**

A comprehensive search yielded 108 records meeting inclusion criteria, involving 178 patients. We identified 46 different IEI according to IUIS 2022 Classification. Combined immunodeficiencies, immune dysregulation disorders, and phagocyte defects were the IEI most frequently associated with HLH. In 75% of cases, HLH preceded the IEI diagnosis, often with an unrecognized history of severe infections. Triggers reflected the specific infection susceptibilities within IEI groups. Liver and central nervous system involvement were less common than in FHL cases. Treatment approaches and outcomes varied, with limited long-term follow-up data, limiting the assessment of therapeutic efficacy across IEI groups.

**Conclusion:**

A comprehensive evaluation encompassing immunological, infectious, and genetic aspects is essential in pediatric-HLH. Relying solely on FHL or EBV susceptibility disorders tests is insufficient, as diverse other IEI can contribute to HLH. Early recognition of HLH as a potential warning sign can guide timely diagnostic investigations and facilitate tailored therapeutic interventions for improved outcomes.

**Systematic review registration:**

https://www.crd.york.ac.uk/prospero/display_record.php?RecordID=371425, PROSPERO, CRD42022371425.

## Introduction

Hemophagocytic lymphohistiocytosis (HLH) is a rare, hyper-acute and potentially life-threatening clinical entity caused by a severe impairment of the immune homeostasis. Previously seen and managed as a single disease, HLH represents a potential clinical expression of several diseases. HLH occurring in patients bearing known mutations in genes related to granule-dependent cytotoxicity are termed “primary” or familial HLH (FHL) (1). According to the International Union of Immunological Societies (IUIS) (2), FHL are Inborn Errors of Immunity (IEI) presenting with HLH as their predominant clinical feature. On the contrary, when HLH is triggered by infections, autoimmune manifestations or malignancy in the absence of specific mutations in FHL-related genes, it is termed “secondary” or “acquired” (1). However, this classification may be considered overly simplistic, since primary HLH are often triggered by infections or other events that activate the immune system as well as secondary HLH might hide unrecognized or unknown genetic causes. Moreover, since HLH both primary and secondary stems from a loss of immune homeostasis, several IEI other than FHL (IEInotFHL) could predispose to HLH. This systematic review aims to characterize HLH in patients with IEInotFHL, especially those in pediatric age. In fact, since the first diagnostic and therapeutic steps for HLH are often taken in the general pediatric setting, pediatricians should be aware of the existence of possible underlying IEI beyond FHL to minimize the potentially fatal risks associated with a missed diagnosis.

## Methods

This systematic review followed the Preferred Reporting Items for Systematic Reviews and Meta-Analyses (PRISMA) recommendations (3). The study protocol was registered as PROSPERO CRD42022371425.

### Definitions

*IEI*: 485 inherited disorders, often due to mutations in a single gene, involving specific impairment of normal development and immune function. IUIS2022 Classification groups IEI into 9 major categories and multiple subgroups based on which part of immune system is impaired (see **Supplemental Data** for complete classification) (2). IUIS2022 Classification also considers an additional group for phenocopies of IEI.

*FHL*: IEI which share HLH as their predominant clinical feature. According to IUIS 2022 Classification FHL can be distinguished in two subgroups based on the presence of hypopigmentation. The first subgroup includes pathogenic variants in PRF1, STX1, UNC13D, STXBP2, FAAP24, SLC7A7, and RHOG, while the second subgroup consists of gene variants associated with Chediak-Higashi syndrome, Griscelli type 2, and, less commonly, Hermansky-Pudlak syndrome types 2 and 10, in addition to the neofunction of CEBPE.

*IEInotFHL*: IEI not belonging to FHL subgroups according to IUIS 2022 Classification.

### Study design and search strategy

On 30th September 2022 an extensive search for publications on HLH associated to IEInotFHL was conducted on PubMed, MEDLINE, EMBASE, and Cochrane Central databases (see **Supplementary Data** for strings). The search string included IEInotFHL previously described as possibly associated to HLH (4–6) and Epstein-Barr Virus (EBV) susceptibility disorders that are not comprised in FHL subgroups according to IUIS 2022 Classification (2). Reference lists were hand-searched for further relevant studies.

### Study eligibility and quality assessment

After duplicates were removed through Rayyan online database (7), screening on title and abstract was conducted by three independent reviewers (W.M.S, S.R and M.F). Discrepancies were discussed until a common decision was reached.

A study was considered eligible when the following criteria were met: at least 5 diagnostic criteria of HLH (8), confirmed diagnosis or high suspicion of IEInotFHL according to IUIS Classification 2022, clinical data report. Records were excluded if they did not describe IEInotFHL, described animal experiments, contained no clinical data, or were not peer reviewed. Phenocopies of IEI were not considered. Non-English records were finally excluded for practical purposes. Quality assessment was performed with Critical Appraisal Tools of the Johanna Briggs Institute (JBI) by two independent reviewers (W.M.S and S.R) (9, 10). No records were excluded because of methodological quality.

### Data extraction, synthesis of results and analysis

All data were extracted by a reviewer (W.M.S) using a Microsoft^®^ Excel^®^ spreadsheet developed by the author team and verified by a second reviewer (S.R.). Common decision was to extract the worst laboratory values. The main characteristics of the included studies have been analyzed and summarized in tables. Quantitative variables are expressed as mean and standard deviations (DS) or median and interquartile range (IQR). GraphPad^®^ Prism 9 was used for statistical analysis. Mann-Whitney test was used to compare non-normal values. Fisher and χ2 tests were used to evaluate differences between groups. The level of significance was set to p<0.05.

## Results

### Study selection

**Figure 1:**
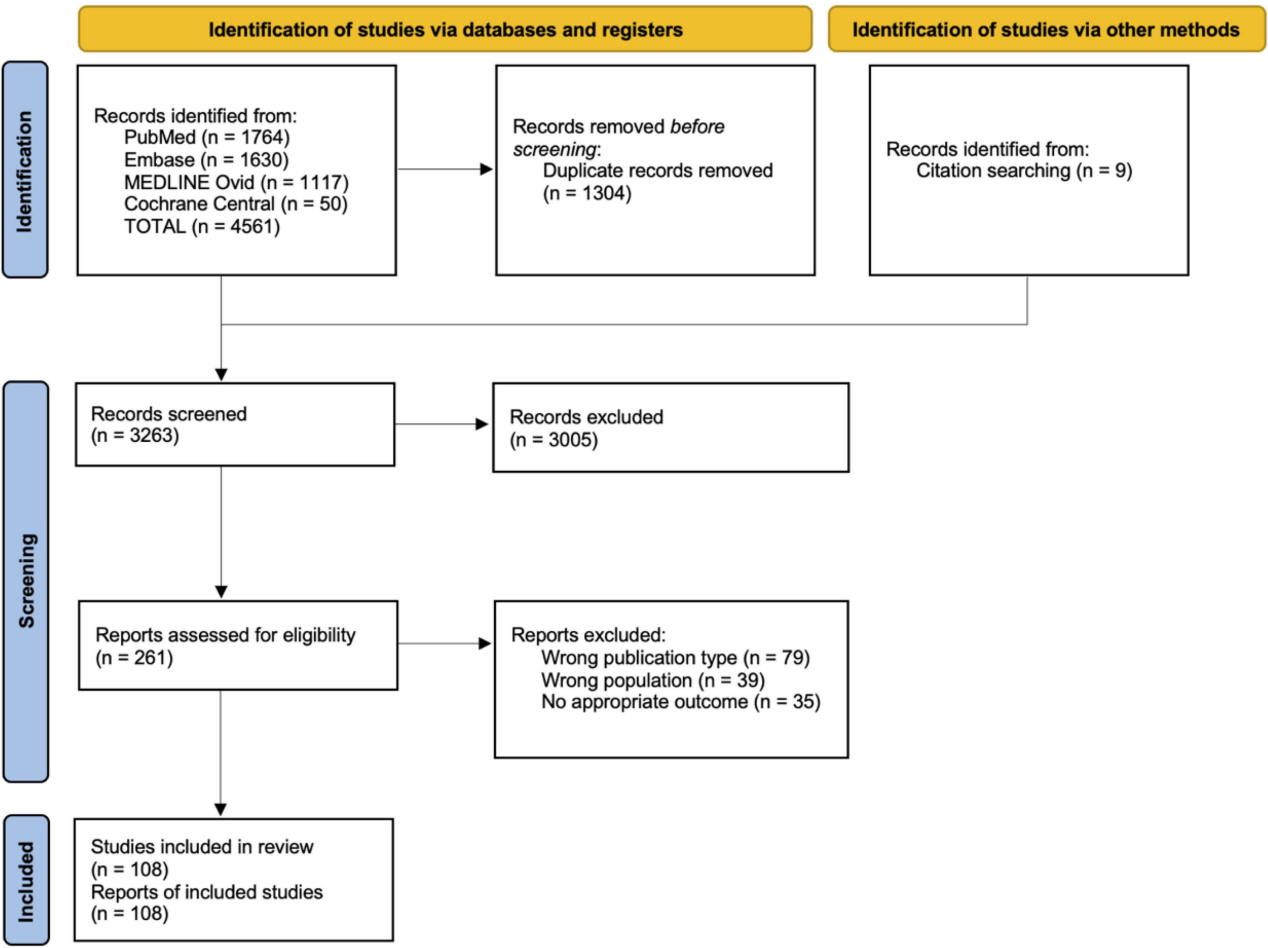
Selected reports flow-chart according to PRISMA Guidelines.

This review included 108 studies (**Figure 1**) for a total of 178 patients (4, 5, 11–116). Through database search and reference lists screening, 4570 records were identified. After 1304 duplicates were removed, 3263 titles/abstracts were screened and subsequently, 261 full-text articles were assessed for eligibility. All details are shown in **Figure 1**.

### Demographics

At HLH onset, 159/178 patients (89%) were aged 0-18 years, 7/159 pediatric patients (4%) experienced HLH within first 30 days of life (IQR 9-23.5 days) (19, 41, 61, 63, 92, 95, 110) and one of them during fetal life (55). The median age at HLH diagnosis, considering only pediatric patients, was 17.5 months (IQR 4-60 months).

Gender was declared or deductible from genetic diagnosis for 149/178 patients (84%) with a M:F ratio 3:1. As expected, gender ratio varied across distinct IEI groups, according with the inheritance pattern. When excluding all X-linked defects, the M:F ratio was established to 1.5:1. Consanguinity between parents was reported in 28/178 patients (16%) while familiarity for IEI or sudden infant death was reported in 30/178 patients (17%).

### HLH in IEInotFHL

The frequencies of HLH diagnostic criteria in selected patients are shown in **Table 1**.

**Table 1:** Clinical and Laboratory data of patients with HLH and IEInotFHL.

HLH-2004 criteria	Prevalence
*Fever*	171/178 (96.06%)
*Splenomegaly*	163/178 (91.57%)
*Ferritin ≥ 500 µg/L*	168/168 (100%)
*sIL2R ≥ 2400 U/mL*	64/78 (82.05%)
*Fibrinogen ≤ 150 mg/dL*	98/129 (75.96%)
*Triglycerides ≥ 265 mg/dL*	126/145 (86.89%)
*Cytopenia 2/3 cell lines*	153/178 (85.95%)
*Hemoglobin ≤9 g/dL (or ≤10 g/dL in first 4 weeks)*	108/116 (93.10%)
*Neutrophils ≤ 1000/µL*	42/83 (50.60%)
*Platelets ≤ 100000/µL*	113/125 (90.40%)
*Hemophagocytosis in bone marrow, liver, spleen*	103/137 (75.18%)
*Reduced NK cytotoxicity*	26/44 (59.09%)
Laboratory	*Median values and IQR [data available]*
*Ferritin (µg/L)*	4847 (1957.75 20816) [168/178]
*sIL2R (U/mL)*	4209.4 (2626.25 9645.56) [168/178]
*Fibrinogen (mg/dL)*	120 (90 170) [129/178]
*Triglycerides (mg/dL)*	364.5 (290 534.5) [145/178]
*Hemoglobin (g/dL)*	7.7 (6.7 8.7) [116/178]
*Neutrophils (cell//µL)*	1240 (360 2530) [83/178]
*Platelets (cell//µL)*	37500 (19750 71000) [125/178]
*ALT (U/L)*	301.5 (100.3 614) [54/178]
*Albumin (g/dL)*	2.3 (2.1 2.4) [12/178]
*Total Bilirubin (mg/dL)*	1.6 (0.96 4.3) [19/178]
*Direct Bilirubin (mg/dL)*	1.3 (0.73 2.93) [13/178]
*D-dimer (mg/L)*	5360 (1113 10520) [10/178]
*INR*	1.67 (1.48 2.10) [21/178]
*LDH (U/L)*	1366.5 (738.5 3247) [39/178]
*CXCL9 (pg/mL)*	27292 (19776 75153.5) [4/178]
*Serum IgG (mg/dL)*	480 (235 1075) [47/178]
*Serum IgA (mg/dL)*	100.5 (30.3 158.3) [46/178]
*Serum IgM (mg/dL)*	53.5 (18.8 181.8) [46/178]
*Serum IgE (KU/L)*	72.5 (27.5 226.3) [14/178]
*CD3+ (cell//µL)*	534 (17 1729) [32/178]
*CD3+CD4+ (cell//µL)*	325 (21 1302) [31/178]
*CD3+CD8+ (cell//µL)*	482 (9 1452) [29/178]
*CD19+ (cell//µL)*	343 (19 1071) [38/178]
*CD56+CD16+ (cell//µL)*	92.5 (10 289) [32/178]

Genetic diagnosis of IEI was available for 149/178 patients (84%) and causative mutations were reported for 115/149 patients (77%). Precise characterization of the genetic investigations has been challenging due to incomplete data and a lack of information regarding the specific timing of these analyses. For the remaining 16% of cases, the diagnosis of IEInotFHL was established without genetic analysis but relied on criteria defined by ESID (European Society for Immunodeficiencies) based on medical history, clinical presentation, and functional laboratory parameters (117). However, for other IEInotFHL, such as those related to innate immunity (12/12 patients, 100%) and autoinflammatory disorders (23/24 patients, 96%), specific diagnosis was only possible through genetic analysis. Otherwise, these conditions would have remained undiagnosed due to the absence of decisive functional tests.

**Figure 2:**
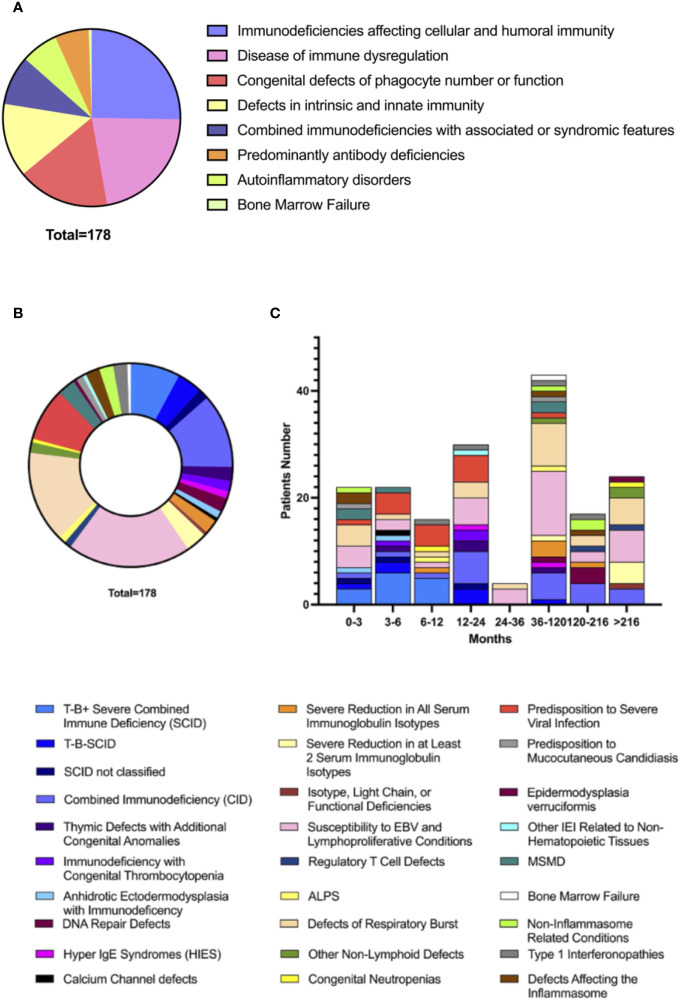
Distribution of HLH in Groups **(A)** of IEInotFHL and Subgroups **(B)** of IEInotFHL according with IUIS 2022 Classification of IEI and distribution of subgroup according to age **(C)**.

Data collection identified 46 different IEI complicated by HLH at the onset or during disease course (See **Supplementary Data** for complete list). All IUIS 2022 IEI major groups were represented, apart from complement defects (**Figure 2**). Most HLH cases identified belonged to groups of IEI affecting both cellular and humoral immunity (45/178, 25%), immune dysregulation disorders (39/178, 22%), defects of phagocyte number or function (30/178, 17%), and defects of intrinsic and innate immunity (24/178, 13%). Recurrence of HLH was reported in 21/178 patients (12%), mainly in intrinsic and innate immunity defects (7/24 vs 14/154, p=0.0048). The distribution of Groups and Subgroups of IEInotFHL is shown in **Figure 2**, according to IUIS 2022 classification (2). (See **Supplementary Data** for complete list of identified IEInotFHL).

### HLH as first sign of IEInotFHL

The age of HLH onset in the different immune defects are detailed in **Figure 2**. Predominantly antibody deficiencies presented with HLH at an older age than all the other groups of IEI (mean values 207.9 ± 203.42 vs 67.64 ± 101.04 months; p=0.0039) while patients with SCID showed the earliest onset of HLH compared to all the other subgroups of IEI (mean values 8.22 ± 12.16 vs 87.04 ± 119.46 months; p<0.0001).

Temporal relation between HLH and IEI diagnosis was available for 172/178 patients (97%). In 127/172 patients (74%) HLH preceded IEI diagnosis. However, when evaluating the medical history prior to the HLH event (available for 98/172 patients; 57%), a history of infections could be identified in 38/127 patients (30%), and in 15/127 (12%) at multiple sites. The most frequent infections were pneumonia 17/127 (13%), recurrent in more than half of the cases (59%), upper respiratory tract infections 11/127 (9%), acute otitis media 7/127 (6%), chronic or recurrent sinusitis 4/127 (3%), sepsis 4/127 (3%), chronic/recurrent muco-cutaneous candidiasis 4/127 (3%), skin or visceral abscesses 3/127 (2%), severe skin infections 2/127 (2%), live strain vaccine viral infections 2/127 (2%), meningoencephalitis and osteomyelitis 1/127 each (1%).

Furthermore, other non-infectious signs or symptoms suggestive of IEI were reported prior to the first episodes of HLH. This included failure to thrive in 10/127 patients (8%) or chronic/recurrent bloody or watery diarrhea in 8/127 patients (6%). One patient was previously suspected for Inflammatory Bowel Disease (IBD) (73). Facial dysmorphisms were described in 5/127 patients (4%) while hematologic anomalies such as persistent/recurrent cytopenia or splenomegaly in 13/127 patients (10%). Additionally, a history of hypogammaglobulinemia was reported in 3/127 patients (2%), although in one case it was likely due to nephrotic syndrome (40, 96, 98). Despite available data are limited, 20/127 patients (16%) had silent clinical history before HLH (See **Supplementary Data** for detailed clinical data).

### HLH laboratory data

Laboratory data referred to each group of IEI according to IUIS 2022 Classification are extensively detailed in the Supplement. No significant differences were found among groups of IEI neither in ferritin values, nor in triglycerides, fibrinogen, hemoglobin, and platelets values (**Table 1**). Conversely, patients with IEI affecting both cellular and humoral immunity had significantly lower sIL2R values than all the other IEInotFHL (mean values 2407.25 ± 1467.54 U/mL vs 8015.04 ± 7049.91 U/mL; p=0.0012). Moreover, patients with defects of intrinsic or innate immunity had significantly higher values of neutrophils (mean values 8448 ± 7818 cell/µL vs 1960± 3266cell/µL; p<0.0001) and aspartate aminotransferase (mean values 3831 ± 4855 U/L vs 1188 ± 2015 U/L; p=0.0273) than all other IEInotFHL. Additional laboratory data are listed in **Table 1**.

### Triggers and clinical signs at onset of HLH

**Figure 3:**
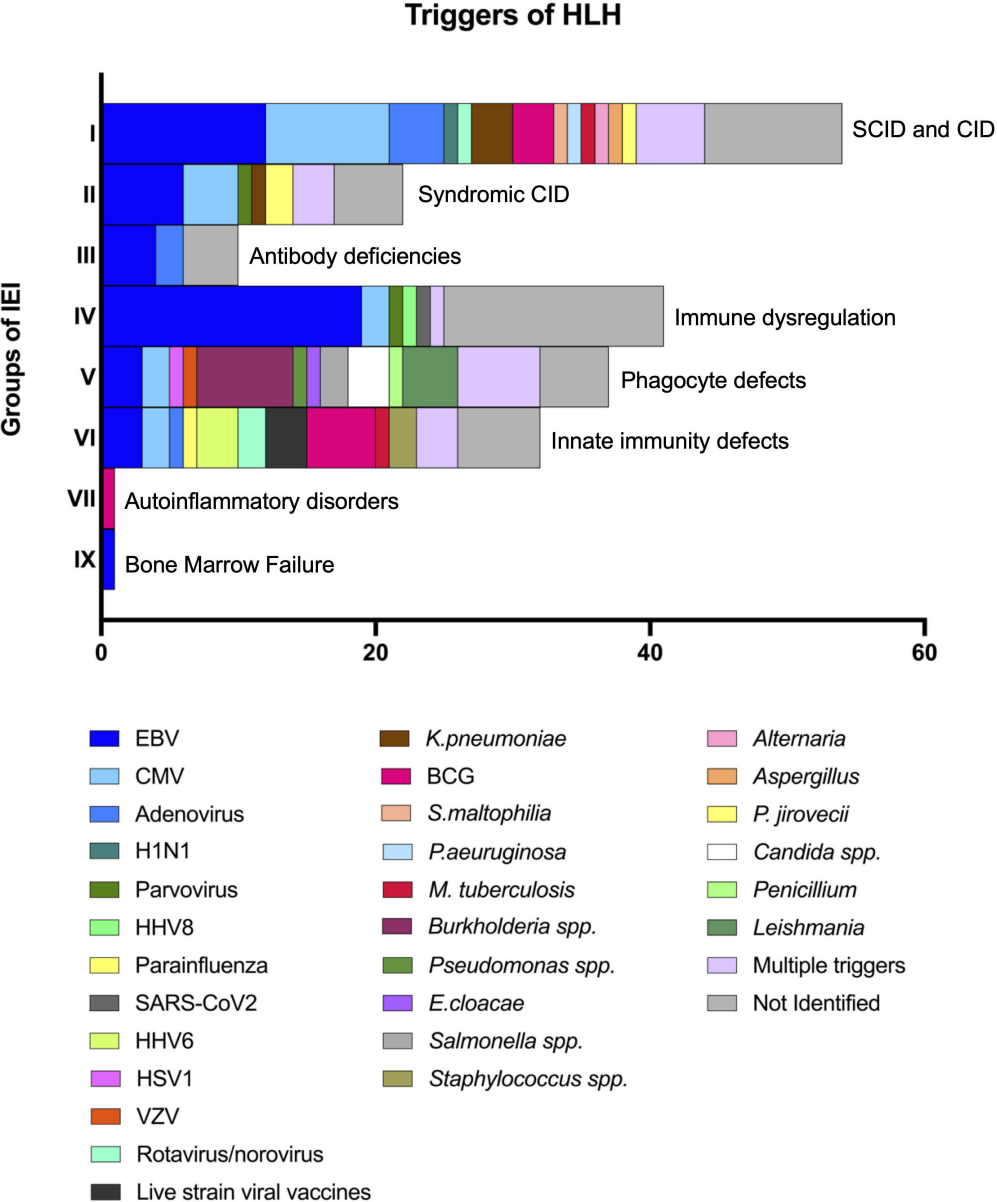
Triggers of HLH in patients with underlying IEInotFHL.

Infectious triggers were reported in 121/178 patients (68%). Multiple concomitant triggers were identified in 21/178 patients (12%). EBV, alone or combined with other pathogens, was found in 48/178 patients (27%), and it was the most frequent trigger of HLH in most groups of IEI (**Figure 3**). However, in 4 patients included in the “susceptibility to EBV and lymphoproliferative disorders” subgroup, HLH was triggered by different viruses (Parvovirus, HHV8, and CMV) (20, 61, 68). Additionally, in 12 patients within the same subgroup a trigger could not be clearly identified after EBV infection was ruled out.

HLH was infrequently triggered by infections in patients with IEI classified as autoinflammatory disorders (e.g., type 1 interferonopathies or defects affecting the inflammasome) when compared to all the others (1/12 vs 120/166, p<0.0001) (25). Selective triggers were identified for specific IEI: fungal infections were more frequent in patients with defects of phagocyte number or function (5/25 vs 5/96, p=0.0167) while BCG was more commonly found in patients with intrinsic or innate immunity disorders (5/19 vs 3/102, p=0.0024). Leishmania was identified only in patients with CGD (3 X-linked and 1 AR-CGD) (4, 62) while live-strain viral vaccine were only in patients with intrinsic or innate immunity disorders (21, 53, 103).

Regardless of infectious trigger, airways were the most frequent site of infection with symptomatic presentation such as cough or dyspnea. Respiratory signs were followed by skin rash (30/178, 17%) and gastrointestinal signs (25/178, 14%). Neurologic signs as consciousness impairment, seizures, ataxia, or focal signs were reported in 21/178 patients (12%) at onset of HLH. Nevertheless, Central Nervous System (CNS) involvement was confirmed by lumbar puncture or brain Magnetic Resonance Imaging (MRI) in 5/21 patients (24%) and 7/21 patients (33%), respectively. Overall, liver involvement, inclusively considering hepatomegaly and transaminases above 100 U/L, was reported in 97/178 patients (54%), while jaundice and liver failure were reported in 5/178 (3%) (30, 38, 63, 70, 80) and 3/178 patients (2%) (45, 61, 80), respectively.

### Treatment and outcome of HLH in IEInotFHL

Treatment data were available for 157/178 patients (88%) and are listed in **Table 2**. HLH-94/04 protocol was set up in 63/157 patients (40%), whereas 21/157 patients (13%) received corticosteroids only, which alone provided resolution in 12/21 patients (57%).

**Table 2:** Treatment of HLH in patients with IEInotFHL.

Treatment	N of patients
**HLH-94/04 protocol**	63/157 (40.12%)
**Corticosteroids only**	21/157 (13.37%)
Specific Treatment	N of patients
**Antimicrobial** Antibiotic Antiviral Antifungal/antiparasitic	58/157 (36.94%) 45/157 (28.66%) 18/157 (11.46%) 17/157 (10.82%)
**Corticosteroids** Dexamethasone MDP Other/not specified	133/157 (84.71%) 97/157 (61.78%) 22/157 (14.01%) 19/157 (12.10%)
**VP16 (Etoposide)**	63/157 (40.12%)
**Cyclosporine**	61/157 (38.85%)
**Anakinra**	11/157 (7%)
**Rituximab**	12/157 (7.64%)
**Emapalumab**	4/157 (2.54%)
**Etanercept**	2/157 (1.27%)
**Azathioprine**	2/157 (1.27%)
**Sirolimus**	2/157 (1.27%)
**Ruxolitinib**	2/157 (1.27%)
**Basiliximab**	1/157 (0.63%)
**Infliximab**	1/157 (0.63%)

Biologic agents or immunosuppressive therapies, as shown in **Table 2**, were used in addition to complete HLH treatment protocol in 8/157 patients (5%) and used as an alternative approach to delay chemotherapy with VP16 in 22/157 patients (14%). Finally, at the time of reports submission, 32/178 patients (18%) underwent Hematopoietic Stem Cell Transplantation (HSCT). More specific details regarding transplantation could not be provided because data were incomplete. Information about type of donor and conditioning regimen were reported for only 14/32 patients (44%) and 13/32 patients (41%), respectively.

Outcome was clearly described for 171/178 patients (96%). At the time of reports submission 84/171 patients (49%) were alive. Age at death was clearly described for 43/171 patients (25%). The leading cause of death were multiorgan failure (22/47, 47%), respiratory failure (16/47, 34%), and sepsis (7/47, 15%). Outcome was significantly worse when the onset of HLH was in the first 12 months (36/57 vs 51/114 patients dead, p=0.0231). Overall mortality was significantly higher among patients with IEI affecting cellular and humoral immunity (CID and SCID) compared to all the other IEI (38/60 vs 49/111 patients dead, p=0.0166). After HSCT, 13/32 patients (41%) died (mean of 57.9 ± 95.8 days). No differences in survival outcome were found between transplanted and not transplanted patients (13/32 vs 74/139 patients dead, p=0.1982). No significative differences were also found between patients with or without CNS involvement (15/25 vs 72/146 patients dead, p=0.3234), or between patients who received etoposide and the ones who did not (35/60 vs 52/111 patients dead, p=0.1516).

## Discussion

Characterization of HLH as a possible clinical manifestation of an underlying IEI is critical to properly manage this life-threatening condition. Moreover, raising awareness among pediatricians and neonatal/pediatric intensivists, who often deal with HLH at onset, is crucial to ensure rapid recognition, appropriate treatment of any underlying IEI, and improved outcomes both during HLH and, even more so, after its remission.

Chinn et al. in 2018 highlighted significant genetic diversity within patients meeting HLH criteria (118). Among 122 subjects enrolled over a 17-year period, genetic testing was conducted on 101 subjects. Notably, whole-exome sequencing (WES) analysis identified IEInotFHL in 14 cases and dysregulated immune activation or proliferation disorders in 8 cases. However, it has long been known that IEInotFHL can predispose to the development of HLH. In 2015 a large survey and literature review on patients with HLH and IEI other than FHL or XLP performed by Bode et al. found that combined immunodeficiencies (CID and SCID) and chronic granulomatous disease (CGD) were the most frequent underlying IEInotFHL (4). Similar conclusions were obtained by Cetinkaya et al. in another single-center study enrolling 28 patients with HLH, between the years 2013 and 2017, in which combined immunodeficiencies represented the most frequent IEInotFHL (5).

In accordance with previous reports (4, 5), we noticed that most cases of HLH occurred in the groups of SCID and CID, in the group of immune dysregulation disorders such as XLP-1 and XLP-2, and in CGD patients. However, all groups in the IUIS classification except complement deficiencies were found to underlie HLH development. Specifically, 46 different IEIs were identified in patients with HLH in this review.

Recognizing the methodology limitation of a systematic review, we acknowledge its non-quantitative nature, impeding the determination of individual IEInotFHL prevalence. Notably, the exclusion of numerous studies exploring the association between EBV susceptibility disorders (e.g., XLP1 or XLP2) and HLH (119–121) was guided by our specific criteria. This exclusion may have potentially led to an underestimation of the significance of each IEInotFHL, especially the well-established EBV susceptibility disorders. Indeed, Gadoury-Levesque et al. proposed that SAP and XIAP deficiency contribute to approximately 15% of genetically confirmed HLH disorders (122). This prevalence aligns with the combination of HLH susceptibility with pigmentary defects and is slightly lower than each of the three common FHL disorders, as indicated by the same study.

Nevertheless, we believe that focusing only on FHL or EBV susceptibility disorders in patients with HLH might be reductive and lead to missed diagnoses of other IEI resulting in increased numbers of complications, sequelae, or death in undiagnosed patients. Therefore, these results suggest that specific functional tests beyond flow cytometric assessment of perforin and NK degranulation activity should be included in first diagnostic steps in all patients, followed by extensive genetic analysis not limited to FHL solely. Genetic investigations, ranging from basic to more in-depth testing, were performed in over 80% of cases, being decisive especially in cohorts of IEInotFHL such as autoinflammatory disorders or innate immunity defects, wherein non-genetic testing was inconclusive. These observations underscore the pivotal role of genetics in discerning intricate immune-related conditions when suspicion is elevated, and conventional immunological assessments remain inconclusive. Additionally, in line with the suggestions of Chinn et al. (118), these findings emphasize the constraints of targeted FHL gene sequencing for the majority of HLH patients, while accentuating the potential of WES to precisely identify other IEI and pinpoint specific therapeutic approaches.

Bode et al. demonstrated that HLH was the initial presentation of IEI in 57% of patients (4). Similar conclusions were obtained by Cetinkaya et al (5). In the present review HLH episodes preceded the diagnosis of IEI in three quarters of patients, often as the first manifestation of the underlying disease. This was also true for SCID, which are usually suspected in first months of life because of severe life-threatening infections. Therefore, it can be speculated that widespread implementation of newborn screening for SCID may also serve as a preventive measure against potentially life-threatening HLH episodes triggered by infections (123–125). On the other hand, a previous history of susceptibility to recurrent or severe infections was reported in one-third of cases diagnosed with IEInotFHL after HLH, but these infections were probably not considered relevant enough to prompt a suspicion of IEI.

The application of clinical screening based on the 10 Jeffrey Modell Foundations warning signs based on infectious disease susceptibility, has greatly promoted knowledge of immunodeficiencies in the last decades. Nevertheless the International Immunology Network emphasized the importance of inflammatory and autoimmune manifestations among the warning signs to reduce the missed diagnoses of IEI (126). In this regard it could be useful to consider HLH as a possible warning sign for IEI and include it among the many outlined by the Jeffrey Modell Foundation.

The present review showed that HLH triggers usually reflect the specific susceptibility to infections of different groups of IEI. Fungal triggers indeed, were more represented among patients with underlying defects of phagocyte number and function while live strain vaccines like BCG were among patients with specific disorders of innate immunity like mendelian susceptibilities to mycobacterial disease. These data reinforce the theory that HLH is often a state of “immune frustration” due to the inability of the immune system to efficiently fight infection and achieve complete clearance or control of the pathogen resulting in a self-sustaining cycle of antigenic stimulation and hyperinflammation. The identification of specific pathogens in the context of an episode of HLH could help direct clinical suspicion to a specific underlying IEInotFHL. For instance, CGD should always be investigated after the identification of *Leishmania* spp., *Candida* spp. or *Burkholderia* spp. in HLH patients, even more so if they have recurrent or persistent HLH. Anyway, in accordance with the literature, viruses were the primary triggers of HLH in almost all groups of IEInotFHL (127). Other viruses were also identified as triggers in patients with EBV-related disorders, as well as EBV was the trigger even in non-EBV-related disorders. Therefore, infectious workup should be as wide as possible and not limited to the detection of EBV.

Regardless of the infectious trigger, children with IEInotFHL may develop HLH in the context of respiratory or gastrointestinal infections more frequently (25%) than those with FHL who rarely show signs of infection at the onset of HLH (128). In contrast to the inflammatory phenotype, liver and CNS involvement was reported more rarely in patients with HLH and underlying IEInotFHL than in FHL, where CNS involvement ranges from 30% to 73% of cases and implies a worse outcome, as confirmed by Amirifar et al (129, 130).

Comparison of data from this review with those in the literature found no significant differences in routine diagnostic markers of HLH compared with secondary HLH or FHL.

Regarding treatment, the data obtained from this review are uneven and not based on long follow-up, making them inconsistent for speculations on therapeutic efficacy in the different IEI groups. However, as expected in view of the high infectious risk, less than half of patients received HLH-1994 or HLH-2004 treatment with chemotherapy. This may partly be attributed to the identification of specific pathogens as trigger of HLH which prompted clinicians to immediately start targeted antimicrobial treatment and delay the application of chemotherapy. In two cases, this approach resolved HLH without additional treatments (32, 47).

Acknowledging the partial limitations of the data, it is critical to point out that the results of chemotherapy were not significantly better. While FHL is a more uniform category, HLH due to IEInotFHL requires subcategorization for tailored therapy, as it does not always involve T-cell activation. In fact, in the North American Consortium for Histiocytosis (NACHO) recommendations, Jordan et al. introduced the term “HLH disease mimics” to refer to all those conditions that, while meeting HLH criteria, would not benefit from immunosuppression (131). Based on the observations from this systematic review, some HLH due to IEInotFHL might actually meet this definition. Thus, although data should be interpreted with caution due to potential sources of bias, our suggestion is that patients with IEInonFHL may not necessarily require comprehensive treatment protocols for HLH.

Therefore, cautious evaluation and monitoring, along with individualized treatment strategies based on underlying IEInotFHL or identified triggers, remain mandatory.

In addition, pathway-specific target therapies are progressively gaining more space in the treatment of HLH at the expense of broad-spectrum etoposide-based chemotherapy, especially for patients with IEI, both because of a better understanding of individual pathogenetic defects (e.g., defects in interferon pathways) and because of greater availability of new molecules that necessarily exert a less global immunosuppressive effect.

Although the overall outcomes did not appear to be significantly influenced by HSCT, it is important to note that these findings should be interpreted with caution due to the lack of long-term follow-up data and insufficient details on conditioning regimens and prophylaxis of graft-versus-host disease (GVHD) in the selected studies. Further research and comprehensive analysis are needed to provide a more conclusive understanding of the impact of treatments and outcomes of HLH in the context of IEI.

The main limitation of this work is the retrospective design of the study which does not allow conclusions about significant differences in laboratory features for specific IEIs or treatment approaches that are often not well described.

## Conclusions

HLH represents a significant and unpredictable clinical challenge for pediatricians and pediatric intensivists who often manage this clinical emergency at its onset. To the best of our knowledge this is the first systematic review about HLH and IEI other than FHL. The data presented within this study suggest that HLH could potentially emerge as a clinical hallmark across various forms of IEInotFHL, often serving as their initial recognizable indicator. Proficiency in discerning HLH indicators and, notably, uncovering the etiology of this potentially fatal condition can notably enhance patient prognoses, extending benefits even beyond HLH remission. The recognition that early detection of underlying genetic origins can reshape patient management in next future is evident. As the genetic landscape unfolds, an appealing transition to personalized approaches emerges, enriching therapeutic options and directing us toward precision interventions, ultimately leading to improved patient outcomes.

## Data availability statement

The original contributions presented in the study are included in the article/**Supplementary Material**. Further inquiries can be directed to the corresponding author.

## Author contributions

SR: Conceptualization, Data curation, Investigation, Methodology, Writing – original draft, Writing – review & editing. WS: Conceptualization, Data curation, Investigation, Methodology, Writing – original draft, Writing – review & editing. LL: Writing – review & editing. CC: Writing – review & editing. FL: Writing – review & editing. DD: Writing – review & editing. MF: Writing – review & editing. LP: Writing – review & editing. ES: Supervision, Validation, Writing – review & editing. GI: Supervision, Validation, Writing – review & editing. MR: Supervision, Validation, Writing – review & editing. CA: Supervision, Validation, Writing – review & editing.

## References

[1] Farquhar JW Claireaux AE . Familial haemophagocytic reticulosis. Arch Dis Childhood. (1952) 27:519. doi: 10.1136/ADC.27.136.519 13008468 PMC1988563

[2] Tangye SG Al-Herz W Bousfiha A Cunningham-Rundles C Franco JL Holland SM . Human inborn errors of immunity: 2022 update on the classification from the international union of immunological societies expert committee. J Clin Immunol. (2022) 42(7):1473–1507. doi: 10.1007/S10875-022-01289-3 PMC9244088 35748970

[3] Page MJ McKenzie JE Bossuyt PM Boutron I Hoffmann TC Mulrow CD . The PRISMA 2020 statement: an updated guideline for reporting systematic reviews. BMJ (2021) 372:n71. doi: 10.1136/BMJ.N71 33782057 PMC8005924

[4] Bode SFN Ammann S Al-Herz W Bataneant M Dvorak CC Gehring S . The syndrome of hemophagocytic lymphohistiocytosis in primary immunodeficiencies: implications for differential diagnosis and pathogenesis. Haematologica. (2015) 100:978–88. doi: 10.3324/HAEMATOL.2014.121608 PMC4486233 26022711

[5] Cetinkaya PG Cagdas D Gumruk F Tezcan I . Hemophagocytic lymphohistiocytosis in patients with primary immunodeficiency. J Pediatr Hematology/Oncology. (2020) 42:E434–9. doi: 10.1097/MPH.0000000000001803 32324696

[6] Canna SW Marsh RA . Pediatric hemophagocytic lymphohistiocytosis. Blood. (2020) 135:1332–43. doi: 10.1182/BLOOD.2019000936 PMC8212354 32107531

[7] Ouzzani M Hammady H Fedorowicz Z Elmagarmid A . Rayyan—a web and mobile app for systematic reviews. Systematic Rev. (2016) 5:210. doi: 10.1186/s13643-016-0384-4 PMC5139140 27919275

[8] Henter JI Horne AC Arico M Egeler RM Filipovich AH Imashuku S . HLH-2004: Diagnostic and therapeutic guidelines for hemophagocytic lymphohistiocytosis. Pediatr Blood cancer. (2007) 48:124–31. doi: 10.1002/PBC.21039 16937360

[9] Munn Z MClinSc SM Lisy K Riitano D Tufanaru C . Methodological guidance for systematic reviews of observational epidemiological studies reporting prevalence and cumulative incidence data. Int J Evidence-Based healthcare. (2015) 13:147–53. doi: 10.1097/XEB.0000000000000054 26317388

[10] Ma LL Wang YY Yang ZH Huang D Weng H Zeng XT . Methodological quality (risk of bias) assessment tools for primary and secondary medical studies: what are they and which is better? Military Med Res. (2020) 7:7. doi: 10.1186/s40779-020-00238-8 PMC7049186 32111253

[11] Agarwal A Sharma S Airun M . Symptomatic primary selective IgM immunodeficiency - B lymphoid cell defect in adult man with secondary HLH syndrome. J Assoc Physicians India. (2016) 64(7):91–93. 27759358

[12] Al-Hammadi S Yahya AM Al-Amri A Shibli A Balhaj GB Tawil MI . Case report: BCG-triggered hemophagocytic lymphohistiocytosis in an infant with X-linked recessive mendelian susceptibility to mycobacterial disease due to a variant of chronic granulomatous disease. Front pediatrics. (2021) 9:687538. doi: 10.3389/fped.2021.687538 PMC8275851 34268280

[13] Alawbathani S Westenberger A Ordonez-Herrera N Al-Hilali M Al Hebby H Alabbas F . Biallelic ZNFX1 variants are associated with a spectrum of immuno-hematological abnormalities. Clin Genet. (2022) 101(2):247–254. doi: 10.1111/cge.14081 34708404

[14] Alsalamah M Roifman CM . Hemophagocytic ymphohistiocytosis associated with ataxia telangiectasia. LymphoSign J. (2017) 4(3):113–116. doi: 10.14785/lymphosign-2017-0007

[15] Alsalamah M Sarpal A Siu VM Gibson P Rupar CA Barton M . Hemophagocytic lymphohistiocytosis in a patient with CD3δ deficiency. LymphoSign J. (2015) 2(4):201–206. doi: 10.14785/lpsn-2015-0006

[16] Arico M Bettinelli A Maccario R Clementi R Bossi G Danesino C . Hemophagocytic lymphohistiocytosis in a patient with deletion of 22q11.2. Am J Med Genet. (1999) 87(4):329–30. doi: 10.1002/(SICI)1096-8628(19991203)87:4<329::AID-AJMG9>3.3.CO;2-D 10588839

[17] Aytekin ES Cagdas; D Tan C Cavdarlj B Bilgic I Tezcan I . Hematopoietic stem cell transplantation complicated with EBV associated hemophagocytic lymphohistiocytosis in a patient with DOCK2 deficiency. Turkish J Pediatrics. (2021) 63:1072–7. doi: 10.24953/turkjped.2021.06.016 35023658

[18] Bajaj P Clement J Bayerl MG Kalra N Craig TJ Ishmael FT . High-grade fever and pancytopenia in an adult patient with common variable immune deficiency. Allergy Asthma Proc. (2014) 35(1):78–82. doi: 10.2500/aap.2014.35.3704 24433602

[19] Barsalou J Blincoe A Fernandez I Dal-Soglio D Marchitto L Selleri S . Rapamycin as an adjunctive therapy for NLRC4 associated macrophage activation syndrome. Front Immunol. (2018) 9:2162. doi: 10.3389/fimmu.2018.02162 30319625 PMC6166634

[20] Bird JA McClain KL Rosenblatt HM Abramson SL Hanson IC . Hemophagocytic lymphohistiocytosis in a patient with x-linked lymphoproliferative disease. Allergy Asthma proceedings : Off J regional state Allergy societies. (2009) 30(4):458–62. doi: 10.2500/aap.2009.30.3259 19772767

[21] Boehmer DFR Koehler LM Magg T Metzger P Rohlfs M Ahlfeld J . A novel complete autosomal-recessive STAT1 LOF variant causes immunodeficiency with hemophagocytic lymphohistiocytosislike hyperinflammation. J Allergy Clin Immunology: In Practice. (2020) 8(9):3102–3111. doi: 10.1016/j.jaip.2020.06.034 PMC9188869 32603902

[22] Burak N Jan N Kessler J Oei E Patel P Feldman S . Diagnosis of GATA2 deficiency in a young woman with hemophagocytic lymphohistiocytosis triggered by acute systemic cytomegalovirus infection. Am J Case Rep. (2021) 22:e927087. doi: 10.12659/AJCR.927087 33684095 PMC7959100

[23] Burns C Cheung A Stark Z Choo S Downie L White S . A novel presentation of homozygous loss-of-function STAT-1 mutation in an infant with hyperinflammation—A case report and review of the literature. J Allergy Clin Immunology: In Practice. (2016) 4(4):777–9. doi: 10.1016/j.jaip.2016.02.015 27117246

[24] Butt FF Mir FF Madasu A Humad H Rana AN . Recurrent infection and immune dysfunction: A case of NCF-2 gene mutation with secondary hemophagocytic lymphohistiocytosis. Dubai Med J. (2022) 5(2):129–132. doi: 10.1159/000521700

[25] Castro CN Rosenzwajg M Carapito R Shahrooei M Konantz M Khan A . NCKAP1L defects lead to a novel syndrome combining immunodeficiency, lymphoproliferation, and hyperinflammation. J Exp Med. (2020) 217(12):e20192275. doi: 10.1084/JEM.20192275 32766723 PMC7526481

[26] Celiksoy MH Ozyavuz Cubuk P Guner SN Yildiran A . A case of ataxia-telangiectasia presented with hemophagocytic syndrome. J Pediatr Hematology/Oncology. (2018) 40(8):e547–e549. doi: 10.1097/MPH.0000000000001134 29620677

[27] Cesaro S Messina C Sainati L Danesino C Arico M . Del 22Q11.2 and Hemophagocytic lymphohistiocytosis: A non-random association [3]. Am J Med Genet. (2003) 116:208–9. doi: 10.1002/ajmg.a.10122 12494446

[28] Chidambaram AC Maulik K Ramamoorthy JG Parameswaran N . A novel mutation of adenosine deaminase causing SCID presenting as hemophagocytic lymphohistiocytosis with acute kidney injury. Br J Haematology. (2020) 191:509–12. doi: 10.1111/bjh.17058 33174709

[29] Cui T Wang Y Wang J Zhang J Gao Z Wang Z . The role of allogeneic hematopoietic stem cell transplantation and Epstein-Barr virus infection on the treatment for child primary hemophagocytic lymphohistiocytosis patients with X-linked lymphoproliferative disease: A rare case report and family survey study. Pediatr Transplantation. (2020) 24:e13635. doi: 10.1111/petr.13635 32011062

[30] De La Varga-Martinez R Mora-Lopez F Garcia-Cuesta D Garrastazul-Sánchez MP Quintero S Rodríguez C . X-linked lymphoproliferative disease type 1 in a patient with the p.Gly93Asp SH2D1A gene mutation and hemophagocytic lymphohistiocytosis. J Pediatr Hematology/Oncology. (2017) 39(8):e483–e485. doi: 10.1097/MPH.0000000000000938 28816794

[31] Dvorak CC Sandford A Fong A Cowan MJ George TI Lewis DB . Maternal T-cell engraftment associated with severe hemophagocytosis of the bone marrow in untreated X-linked severe combined immunodeficiency. J Pediatr Hematology/Oncology. (2008) 30(5):396–400. doi: 10.1097/MPH.0b013e318168e7a0 18458578

[32] Eng V Zomorodian TJ Samant SA Maarup TJ Sheikh J . Signal transducer and activator of transcription 1 gain-of-function with refractory hemophagocytic lymphohistiocytosis. Ann allergy Asthma immunology : Off Publ Am Coll Allergy Asthma Immunol. (2020) 125:605–607.e1. doi: 10.1016/J.ANAI.2020.06.042 32629017

[33] Escaron C Ralph E Bibi S Visser J Aricò M Rao K . Diagnosis of HLH: two siblings, two distinct genetic causes. Clin Exp Immunol. (2022) 207:205–7. doi: 10.1093/cei/uxab019 PMC8982960 35020838

[34] Gera A Misra A Tiwari A Singh A Mehndiratta S . A hungry Histiocyte, altered immunity and myriad of problems: Diagnostic challenges for Pediatric HLH. Int J Lab Hematology. (2021) 43. doi: 10.1111/ijlh.13626 34118134

[35] Gothe F Hatton CF Truong L Klimova Z Kanderova V Fejtkova M . A novel case of homozygous interferon alpha/beta receptor alpha chain (IFNAR1) deficiency with hemophagocytic lymphohistiocytosis. Clin Infect Diseases. (2022) 74(1):136–139. doi: 10.1093/cid/ciaa1790 PMC8752251 33252644

[36] Greil J Verga-Falzacappa MV Echner NE Behnisch W Bandapalli OR Pechanska P . Mutating heme oxygenase-1 into a peroxidase causes a defect in bilirubin synthesis associated with microcytic anemia and severe hyperinflammation. Haematologica. (2016) 101(11):e436–e439. doi: 10.3324/haematol.2016.147090 27662012 PMC5394876

[37] Grunebaum E Zhang J Dadi H Roifman CM . Haemophagocytic lymphohistiocytosis in X-linked severe combined immunodeficiency. Br J Haematology. (2000) 108:834–7. doi: 10.1046/j.1365-2141.2000.01923.x 10792291

[38] Halasa NB Whitlock JA McCurley TL Smith JA Zhu Q Ochs H . Fatal hemophagocytic lymphohistiocytosis associated with epstein-barr virus infection in a patient with a novel mutation in the signaling lymphocytic activation moleculeassociated protein. Clin Infect Diseases. (2003) 37(10):e136–41. doi: 10.1086/379126 14583885

[39] Han SP Lin YF Weng HY Tsai SF Fu LS . A novel BTK gene mutation in a child with atypical X-linked agammaglobulinemia and recurrent hemophagocytosis: A case report. Front Immunol. (2019) 10:1953. doi: 10.3389/fimmu.2019.01953 31481959 PMC6711359

[40] Harnisch E Buddingh EP Thijssen PE Brooks AS Driessen GJ Kersseboom R . Hematopoietic stem cell transplantation in a patient with ICF2 syndrome presenting with EBV-induced hemophagocytic lymphohystiocytosis. Transplantation. (2016) 100(7):e35–6. doi: 10.1097/TP.0000000000001210 27326813

[41] Higuchi T Izawa K Miyamoto T Honda Y Nishiyama A Shimizu M . An efficient diagnosis: A patient with X-linked inhibitor of apoptosis protein (XIAP) deficiency in the setting of infantile hemophagocytic lymphohistiocytosis was diagnosed using high serum interleukin-18 combined with common laboratory parameters. Pediatr Blood Cancer. (2022) 69(8):e29606. doi: 10.1002/pbc.29606 35187790

[42] Honda K Kanegane H Eguchi M Kimura H Morishima T Masaki K . Large deletion of the X-linked lymphoproliferative disease gene detected by fluorescence. Situ hybridization. Am J Hematology. (2000) 64(2):128–32. doi: 10.1002/(sici)1096-8652(200006)64:2<128::aid-ajh11>3.0.co;2-# 10814994

[43] Horneff G Rhouma A Weber C Lohse P . Macrophage activation syndrome as the initial manifestation of tumour necrosis factor receptor 1-associated periodic syndrome (TRAPS). Clin Exp Rheumatol. (2013) 31(3 Suppl 77):99–102. 24064022

[44] Hoshino T Kanegane H Doki N Irisawa H Sakura T Nojima Y . X-linked lymphoproliferative disease in an adult. Int J Hematology. (2005) 82(1):55–8. doi: 10.1532/IJH97.05020 16105760

[45] Hugle B Astigarraga I Henter JI Porwit-Macdonald A Meindl A Schuster V . Simultaneous manifestation of fulminant infectious mononucleosis with haemophagocytic syndrome and B-cell lymphoma in X-linked lymphoproliferative disease. Eur J Pediatrics. (2007) 166(6):589–93. doi: 10.1007/s00431-006-0290-1 17058098

[46] Imashuku S Miyagawa A Chiyonobu T Ishida H Yoshihara T Teramura T . Epstein-Barr virus-associated T-lymphoproliferative disease with hemophagocytic syndrome, followed by fatal intestinal B lymphoma in a young adult female with WHIM syndrome. Ann Hematology. (2002) 81(8):470–3. doi: 10.1007/s00277-002-0489-9 12224006

[47] Jain G Kalra S Sharma S Kumar Vasnik G Gupta R . Hemophagocytic lymphohistiocytosis in a child with chronic granulomatous disease: A rare complication of a rare disorder. Med journal Armed Forces India. (2022) 78:99–102. doi: 10.1016/J.MJAFI.2018.11.012 35035051 PMC8737091

[48] Jiang MY Guo X Sun SW Li Q Zhu YP . Successful allogeneic hematopoietic stem cell transplantation in a boy with X-linked inhibitor of apoptosis deficiency presenting with hemophagocytic lymphohistiocytosis: A case report. Exp Ther Med. (2016) 12(3):1341–1344. doi: 10.3892/etm.2016.3498 PMC4998177 27602064

[49] Kashiwagi Y Kawashima H Sato S Ioi H Amaha M Takekuma K . Virological and immunological characteristics of fatal virus-associated haemophagocytic syndrome (VAHS). Microbiol Immunol. (2007) 51(1):53–62. doi: 10.1111/j.1348-0421.2007.tb03890.x 17237599

[50] Klemann C Ammann S Heizmann M Fuchs S Bode SF Heeg M . Hemophagocytic lymphohistiocytosis as presenting manifestation of profound combined immunodeficiency due to an ORAI1 mutation. J Allergy Clin Immunol. (2017) 140(6):1721–1724. doi: 10.1016/j.jaci.2017.05.039 PMC5723226 28633876

[51] Kuijpers TW Baars PA Aan De Kerk DJ Jansen MH Dors N van Lier RA . Common variable immunodeficiency and hemophagocytic features associated with a FAS gene mutation. J Allergy Clin Immunol. (2011) 127(6):1411–4.e2. doi: 10.1016/j.jaci.2011.01.046 21450335

[52] Lam MT Coppola S Krumbach OHF Prencipe G Insalaco A Cifaldi C . A novel disorder involving dyshematopoiesis, inflammation, and HLH due to aberrant CDC42 function. J Exp Med. (2019) 216(12):2778–2799. doi: 10.1084/jem.20190147 PMC6888978 31601675

[53] Le Voyer T Sakata S Tsumura M Khan T Esteve-Sole A Al-Saud BK . Genetic, immunological, and clinical features of 32 patients with autosomal recessive STAT1 deficiency. J Immunol. (2021) 207(1):133–152. doi: 10.4049/jimmunol.2001451 PMC8702442 34183371

[54] Lekbua A Ouahed J OConnell AE Kahn SA Goldsmith JD Imamura T . Risk-factors associated with poor outcomes in VEO-IBD secondary to XIAP deficiency: A case report and literature review. J Pediatr Gastroenterol Nutr. (2019) 69(1):e13–e18. doi: 10.1097/MPG.0000000000002297 31232887 PMC6607918

[55] Liang J Alfano DN Squires JE Riley MM Parks WT Kofler J . Novel NLRC4 mutation causes a syndrome of perinatal autoinflammation with hemophagocytic lymphohistiocytosis, hepatosplenomegaly, fetal thrombotic vasculopathy, and congenital anemia and ascites. Pediatr Dev pathology : Off J Soc Pediatr Pathol Paediatric Pathol Society. (2017) 20:498–505. doi: 10.1177/1093526616686890 28403691

[56] Liang JH Zhu HY Xu DM Wang L Wang Y Qiao C . A new SH2D1A mutation in a female adult XLP disease with hemophagocytic lymphohistiocytosis and NK-cell leukemia. Ann Hematology. (2019) 98(12):2829–2831. doi: 10.1007/s00277-019-03810-y 31758261

[57] Loganathan A Munirathnam D Sundaram B . X-linked lymphoproliferative disease (XLP1) presenting as non-epstein barr virus (EBV) — Related hemophagocytic lymphohistiocytosis (HLH). Indian Pediatrics. (2020) 57(11):1077–1078. doi: 10.1007/s13312-020-2043-z 33231181

[58] Lougaris V Baronio M Castagna A Tessarin G Rossi S Gazzurelli L . Paediatric MAS/HLH caused by a novel monoallelic activating mutation in p110δ. Clin Immunol. (2020) 219:108543. doi: 10.1016/j.clim.2020.108543 32681977

[59] Maignan M Verdant C Bouvet GF Van Spall M Berthiaume Y . Undiagnosed Chronic Granulomatous Disease, Burkholderia cepacia complex Pneumonia, and Acquired Hemophagocytic Lymphohistiocytosis: A Deadly Association. Case Rep Pulmonology. (2013) 2013:874197. doi: 10.1155/2013/874197 PMC3766577 24058739

[60] Malkan UY Gunes G Aslan T Etgul S Aydin S Buyukasik Y . Common variable immune deficiency associated hodgkins lymphoma complicated with EBV-linked hemophagocytic lymphohistiocytosis: A case report. Int J Clin Exp Med. (2015) 8(8):14203–6. PMC4613081 26550396

[61] Marsh RA Madden L Kitchen BJ Mody R McClimon B Jordan MB . XIAP deficiency: a unique primary immunodeficiency best classified as X-linked familial hemophagocytic lymphohistiocytosis and not as X-linked lymphoproliferative disease. Blood. (2010) 116:1079–82. doi: 10.1182/BLOOD-2010-01-256099 PMC2938130 20489057

[62] Martin A Marques L Soler-Palacin P Caragol I Hernandez M Figueras C . Visceral leishmaniasis associated hemophagocytic syndrome in patients with chronic granulomatous disease. Pediatr Infect Dis J. (2009) 28(8):753–4. doi: 10.1097/INF.0b013e31819c6f3a 19633526

[63] Marzollo A Conti F Rossini L Rivalta B Leonardi L Tretti C . Neonatal manifestations of chronic granulomatous disease: MAS/HLH and necrotizing pneumonia as unusual phenotypes and review of the literature. J Clin Immunol. (2022) 42:299–311. doi: 10.1007/s10875-021-01159-4 34718934

[64] Mischler M Fleming GM Shanley TP Madden L Levine J Castle V . Epstein-Barr virus-induced hemophagocytic lymphohistiocytosis and X-linked lymphoproliferative disease: A mimicker of sepsis in the Pediatric Intensive Care Unit. Pediatrics. (2007) 119(5):e1212–8. doi: 10.1542/peds.2006-1534 17403820

[65] Ozturk C Sutcuoglu S Atabay B Berdeli A . X-linked agammaglobulinemia presenting with secondary hemophagocytic syndrome: A case report. Case Rep Med. (2013) 2013:742795. doi: 10.1155/2013/742795 23424595 PMC3568855

[66] Pachlopnik Schmid JM Junge SA Hossle JP Schneider EM Roosnek E Seger RA . Transient hemophagocytosis with deficient cellular cytotoxicity, monoclonal immunoglobulin M gammopathy, increased T-cell numbers, and hypomorphic NEMO mutation. Pediatrics. (2006) 117(5):e1049–56. doi: 10.1542/peds.2005-2062 16636116

[67] Parekh C Hofstra T Church JA Coates TD . Hemophagocytic lymphohistiocytosis in children with chronic granulomatous disease. Pediatr Blood cancer. (2011) 56:460–2. doi: 10.1002/PBC.22830 21225928

[68] Pasic S Cupic M Lazarevic I . HHV-8-related hemophagocytic lymphohistiocytosis in a boy with XLP phenotype. J Pediatr Hematology/Oncology. (2012) 34(6):467–71. doi: 10.1097/MPH.0b013e3182375372 22258354

[69] Pasic S Micic D Kuzmanovic M . Epstein-Barr virus-associated haemophagocytic lymphohistiocytosis in Wiskott-Aldrich syndrome. Acta Paediatrica Int J Paediatrics. (2003) 92(7):859–61. doi: 10.1080/08035250310003631 12892170

[70] Patiroglu T Haluk Akar H van den Burg M Unal E Akyildiz BN Tekerek NU . X-linked severe combined immunodeficiency due to a novel mutation complicated with hemophagocytic lymphohistiocytosis and presented with invagination: A case report. Eur J Microbiol Immunol. (2014) 4(3):174–6. doi: 10.1556/eujmi-d-14-00019 PMC4160797 25215194

[71] Prader S Felber M Volkmer B Trück J Schwieger-Briel A Theiler M . Life-threatening primary varicella zoster virus infection with hemophagocytic lymphohistiocytosis-like disease in GATA2 haploinsufficiency accompanied by expansion of double negative T-lymphocytes. Front Immunol. (2018) 9:2766. doi: 10.3389/fimmu.2018.02766 30564229 PMC6289061

[72] Prader S Ritz N Baleydier F Andre MC Stähli N Schmid K . X-linked lymphoproliferative disease mimicking multisystem inflammatory syndrome in children—A case report. Front Pediatrics. (2021) 9:691024. doi: 10.3389/fped.2021.691024 PMC8369030 34414143

[73] Qiu KY Liao XY Wu RH Huang K Fang JP Zhou DH . X-linked hyper-igM syndrome: A phenotype of crohns disease with hemophagocytic lymphohistiocytosis. Pediatr Hematol Oncol. (2017) 34(8):428–434. doi: 10.1080/08880018.2017.1409301 29303623

[74] Razaghian A Parvaneh L Delkhah M Abbasi A Sadeghirad P Shahrooei M . Bacillus CalmetteGuerin (BCG)-associated hemophagocytic lymphohistiocytosis in the setting of IFN-γR1 deficiency: A diagnostic dilemma. eJHaem. (2020) 1(1):334–337. doi: 10.1002/jha2.5 PMC9175834 35847695

[75] Ren Y Xiao F Cheng F Huang X Li J Wang X . Whole exome sequencing reveals a novel LRBA mutation and clonal hematopoiesis in a common variable immunodeficiency patient presented with hemophagocytic lymphohistiocytosis. Exp Hematol Oncol. (2021) 10:38. doi: 10.1186/s40164-021-00229-y 34120644 PMC8201866

[76] Ricci S Romano F Nieddu F Picard C Azzari C . OL-EDA-ID syndrome: A novel hypomorphic NEMO mutation associated with a severe clinical presentation and transient HLH. J Clin Immunol. (2017) 37(1):7–11. doi: 10.1007/s10875-016-0350-x PMC5226985 27838798

[77] Rossi-Semerano L Hermeziu B Fabre M Kone-Paut I . Macrophage activation syndrome revealing familial mediterranean fever. Arthritis Care Res. (2011) 63(5):780–3. doi: 10.1002/acr.20418 21557533

[78] Spergel AR Walkovich K Price S Niemela JE Wright D Fleisher TA . Autoimmune lymphoproliferative syndrome misdiagnosed as hemophagocytic lymphohistiocytosis. Pediatrics. (2013) 132(5):e1440-4. doi: 10.1542/peds.2012-2748 24101757 PMC3813387

[79] Salzer E Daschkey S Choo S Gombert M Santos-Valente E Ginzel S . Combined immunodeficiency with life-threatening EBV-associated lymphoproliferative disorder in patients lacking functional CD27. Haematologica. (2013) 98(3):473–8. doi: 10.3324/haematol.2012.068791 PMC3659923 22801960

[80] Schaballie H Renard M Vermylen C Scheers I Revencu N Regal L . Misdiagnosis as asphyxiating thoracic dystrophy and CMV-associated haemophagocytic lymphohistiocytosis in Shwachman-Diamond syndrome. Eur J Pediatrics. (2013) 172(5):613–22. doi: 10.1007/s00431-012-1908-0 23315050

[81] Scheffler-Mendoza SC Yamazaki-Nakashimada MA Olaya-Vargas A Morin-Contreras A Juárez-Echenique JC Alcántara-Ortigoza MA . Successful stem cell transplantation in a child with chronic granulomatous disease associated with contiguous gene deletion syndrome and complicated by macrophage activation syndrome. Clin Immunol. (2014) 154(2):112–5. doi: 10.1016/j.clim.2014.07.004 25063445

[82] Schmid I Reiter K Schuster F Wintergerst U Meilbeck R Nicolai T . Allogeneic bone marrow transplantation for active Epstein-Barr virus-related lymphoproliferative disease and hemophagocytic lymphohistiocytosis in an infant with severe combined immunodeficiency syndrome. Bone Marrow Transplantation. (2002) 29(6):519–21. doi: 10.1038/sj.bmt.1703396 PMC7091774 11960273

[83] Schultz KAP Neglia JP Smith AR Ochs HD Torgerson TR Kumar A . Familial hemophagocytic lymphohistiocytosis in two brothers with X-linked agammaglobulinemia. Pediatr Blood Cancer. (2008) 51(2):293–5. doi: 10.1002/pbc.21573 18421721

[84] Shadur B Abuzaitoun O NaserEddin A Even-Or E Zaidman I Stepensky P . Management of XLP-1 and ITK deficiency: The challenges posed by PID with an unpredictable spectrum of disease manifestations. Clin Immunol. (2019) 198:39–45. doi: 10.1016/j.clim.2018.12.016 30572125

[85] Shahin T Mayr D Shoeb MR Kuehn HS Hoeger B Giuliani S . Identification of germline monoallelic mutations in IKZF2 in patients with immune dysregulation. Blood Advances. (2022) 6(7):2444–2451. doi: 10.1182/bloodadvances.2021006367 PMC9006292 34920454

[86] Sheth J Patel A Shah R Bhavsar R Trivedi S Sheth F . Rare cause of Hemophagocytic Lymphohistiocytosis due to mutation in PRF1 and SH2D1A genes in two children - A case report with a review. BMC Pediatrics. (2019) 19(1):73. doi: 10.1186/s12887-019-1444-4 30849948 PMC6407181

[87] Shi B Chen M Xia Z Xiao S Tang W Qin C . Hemophagocytic syndrome associated with Mycobacterium bovis in a patient with X-SCID: A case report. BMC Infect Diseases. (2020) 20(1):711. doi: 10.1186/s12879-020-05421-9 32993535 PMC7525942

[88] Seidel MG . CD27: A new player in the field of common variable immunodeficiency and EBV-associated lymphoproliferative disorder? J Allergy Clin Immunol. (2012) 129(4):1175. doi: 10.1016/j.jaci.2012.01.053 22365582

[89] Sieni E Cetica V Piccin A Gherlinzoni F Sasso FC Rabusin M . Familial hemophagocytic lymphohistiocytosis may present during adulthood: Clinical and genetic features of a small series. PloS One. (2012) 7(9):e44649. doi: 10.1371/journal.pone.0044649 22970278 PMC3436758

[90] Sirinavin S Techasaensiri C Pakakasama S Vorachit M Pornkul R Wacharasin R . Hemophagocytic syndrome and Burkholderia cepacia splenic microabscesses in a child with chronic granulomatous disease. Pediatr Infect Dis J. (2004) 23(9):882–4. doi: 10.1097/01.inf.0000137565.23501.03 15361735

[91] Spinner MA Ker JP Stoudenmire CJ Fadare O Mace EM Orange JS . GATA2 deficiency underlying severe blastomycosis and fatal herpes simplex virus-associated hemophagocytic lymphohistiocytosis. J Allergy Clin Immunol. (2016) 137(2):638–40. doi: 10.1016/j.jaci.2015.07.043 PMC4747814 26395816

[92] Squire JD Vazquez SN Chan A Smith ME Chellapandian D Vose L . Case report: Secondary hemophagocytic lymphohistiocytosis with disseminated infection in chronic granulomatous disease—A serious cause of mortality. Front Immunol. (2020) 11:581475. doi: 10.3389/fimmu.2020.581475 33362767 PMC7756012

[93] Staines-Boone AT Deswarte C Montoya EV Sánchez-Sánchez LM García Campos JA Muñiz-Ronquillo T . Multifocal recurrent osteomyelitis and hemophagocytic lymphohistiocytosis in a boy with partial dominant IFN-γR1 deficiency: Case report and review of the literature. Front Pediatrics. (2017) 5:75. doi: 10.3389/fped.2017.00075 PMC5413492 28516082

[94] Stepensky P Weintraub M Yanir A Revel-Vilk S Krux F Huck K . IL-2-inducible T-cell kinase deficiency: Clinical presentation and therapeutic approach. Haematologica. (2011) 96(3):472–6. doi: 10.3324/haematol.2010.033910 PMC3046282 21109689

[95] Suzuki N Morimoto A Ohga S Kudo K Ishida Y Ishii E . Characteristics of hemophagocytic lymphohistiocytosis in neonates: A nationwide survey in Japan. J Pediatrics. (2009) 155(2):235–8.e1. doi: 10.1016/j.jpeds.2009.02.050 19446847

[96] Szczawinska-Poplonyk A Ploski R Bernatowska E Pac M . A novel CDC42 mutation in an 11-year old child manifesting as syndromic immunodeficiency, autoinflammation, hemophagocytic lymphohistiocytosis, and Malignancy: A case report. Front Immunol. (2020) 11:318. doi: 10.3389/fimmu.2020.00318 32231661 PMC7082228

[97] Tesi B Sieni E Neves C Romano F Cetica V Cordeiro AI . Hemophagocytic lymphohistiocytosis in 2 patients with underlying IFN-γ receptor deficiency. J Allergy Clin Immunol. (2015) 135(6):1638–41. doi: 10.1016/j.jaci.2014.11.030 25592983

[98] Triebwasser MP Barrett DM Bassiri H Bunin N Elgarten C Freedman J . Combined use of emapalumab and ruxolitinib in a patient with refractory hemophagocytic lymphohistiocytosis was safe and effective. Pediatr Blood Cancer. (2021) 68(7):e29026. doi: 10.1002/pbc.29026 33754483 PMC9269994

[99] Tucci F Gallo V Barzaghi F Ferrua F Migliavacca M Calbi V . Emapalumab treatment in an ADA-SCID patient with refractory hemophagocytic lymphohistiocytosis-related graft failure and disseminated bacillus Calmette-Guerin infection. Haematologica. (2021) 106:641–6. doi: 10.3324/HAEMATOL.2020.255620 PMC7849754 32817285

[100] Uslu N Demir H Balta G Saltik-Temizel IN Ozen H Gürakan F . Hemophagocytic syndrome in a child with severe Crohns disease and familial Mediterranean fever. J Crohns Colitis. (2010) 4(3):341–4. doi: 10.1016/j.crohns.2009.12.005 21122524

[101] Valentine G Thomas TA Nguyen T Lai YC . Chronic granulomatous disease presenting as hemophagocytic lymphohistiocytosis: A case report. Pediatrics. (2014) 134(6):e1727–30. doi: 10.1542/peds.2014-2175 25422023

[102] Van Montfrans JM Rudd E Van Corput LD Henter JI Nikkels P Wulffraat N . Fatal hemophagocytic lymphohistiocytosis in X-linked chronic granulomatous disease associated with a perforin gene variant. Pediatr Blood cancer. (2009) 52:527–9. doi: 10.1002/PBC.21851 19058215

[103] Vavassori S Chou J Faletti LE Haunerdinger V Opitz L Joset P . Multisystem inflammation and susceptibility to viral infections in human ZNFX1 deficiency. J Allergy Clin Immunol. (2021) 148(2):381–393. doi: 10.1016/j.jaci.2021.03.045 PMC8569286 33872655

[104] Vieth S Ammann S Schwarz K Härtel C Schultz C Lehmberg K . Clinical phenotype and functional analysis of a rare XIAP/BIRC4 mutation. Klinische Padiatrie. (2013) 225(6):343–6. doi: 10.1055/s-0033-1355393 24166087

[105] Vignesh P Anjani G Kumrah R Singh A Mondal S Nameirakpam J . Features of hemophagocytic lymphohistiocytosis in infants with severe combined immunodeficiency: Our experience from chandigarh, North India. Front Immunol. (2022) 13:867753. doi: 10.3389/FIMMU.2022.867753 35812426 PMC9260510

[106] Voeten M Maes P Wojciechowski M Vandenbossche L Meyts I Ceulemans B . Extremely elevated cerebrospinal fluid protein levels in a child with neurologic symptoms: Beware of haemophagocytic lymphohistiocytosis. Eur J Paediatric Neurology. (2014) 18(3):427–9. doi: 10.1016/j.ejpn.2013.11.012 24433830

[107] Wegehaupt O Groß M Wehr C Marks R Schmitt-Graeff A Uhl M . TIM-3 deficiency presenting with two clonally unrelated episodes of mesenteric and subcutaneous panniculitis-like T-cell lymphoma and hemophagocytic lymphohistiocytosis. Pediatr Blood Cancer. (2020) 67(6):e28302. doi: 10.1002/pbc.28302 32285995

[108] Wei A Ma H Zhang L Li Z Zhang Q Wang D . Hemophagocytic lymphohistiocytosis resulting from a cytokine storm triggered by septicemia in a child with chronic granuloma disease: A case report and literature review. BMC Pediatrics. (2020) 20(1):100. doi: 10.1186/s12887-020-1996-3 32126983 PMC7053071

[109] White S Mowrer MC Jesudas R Keles ES Roberts JC . Hemophagocytic lymphohistiocytosis in a patient with hyper IgE syndrome. Pediatr Blood Cancer. (2019) 66(10):e27894. doi: 10.1002/pbc.27894 31259457

[110] Yang X Hoshino A Taga T Kunitsu T Ikeda Y Yasumi T . A female patient with incomplete hemophagocytic lymphohistiocytosis caused by a heterozygous XIAP mutation associated with non-random X-chromosome inactivation skewed towards the wild-type XIAP allele. J Clin Immunol. (2015) 35(3):244–8. doi: 10.1007/s10875-015-0144-6 25744037

[111] Yang X Wada T Imadome KI Nishida N Mukai T Fujiwara M . Characterization of Epstein-Barr virus (EBV)-infected cells in EBV-associated hemophagocytic lymphohistiocytosis in two patients with X-linked lymphoproliferative syndrome type 1 and type 2. Herpesviridae. (2012) 3(1):1. doi: 10.1186/2042-4280-3-1 22325832 PMC3298713

[112] Yao J Gu H Mou W Chen Z Ma J Ma H . Various phenotypes of LRBA gene with compound heterozygous variation: A case series report of pediatric cytopenia patients. Int J immunopathology Pharmacol. (2022) 36:3946320221125591. doi: 10.1177/03946320221125591 PMC9465590 36074705

[113] Zhang Q Ma H Ma J Wang D Zhao Y Wang T . Clinical and genetic analysis of immunodeficiency-related diseases associated with PIK3CD mutations. Pediatr Invest. (2018) 2(4):257–262. doi: 10.1002/ped4.12101 PMC7331349 32851276

[114] Zheng F Li J Zha H Zhang J Zhang Z Cheng F . ITK gene mutation: Effect on survival of children with severe hemophagocytic lymphohistiocytosis. Indian J Pediatrics. (2016) 83(11):1349–1352. doi: 10.1007/s12098-016-2079-1 27056244

[115] Zhou S Ma H Gao B Fang G Zeng Y Zhang Q . Characterization of a novel disease-causing mutation in exon 1 of SH2D1A gene through amplicon sequencing: A case report on HLH. BMC Med Genet. (2017) 18(1):15. doi: 10.1186/s12881-017-0376-9 28196537 PMC5310059

[116] Zhou Z Zondag T Hermans M van Hagen PM van Laar JAM . Hemophagocytic lymphohistiocytosis in activated PI3K delta syndrome: An illustrative case report. J Clin Immunol. (2021) 41(7):1656–1659. doi: 10.1007/s10875-021-01080-w PMC8193594 34115277

[117] ESID . European Society for Immunodeficiencies . Available online at: https://esid.org/Working-Parties/Registry-Working-Party/Diagnosis-criteria (Accessed March 7, 2023).

[118] Chinn IK Eckstein OS Peckham-Gregory EC Goldberg BR Forbes LR Nicholas SK . Genetic and mechanistic diversity in pediatric hemophagocytic lymphohistiocytosis. Blood. (2018) 132:89–100. doi: 10.1182/blood-2017-11-814244 29632024 PMC6034641

[119] Booth C Gilmour KC Veys P . X-linked lymphoproliferative disease due to SAP/SH2D1A deficiency: a multicenter study on the manifestations, management and outcome of the disease. Blood. (2011) 117:53–62. doi: 10.1182/blood-2010-06-284935 20926771 PMC3374620

[120] Yang L Booth C Speckmann C Seidel MG Worth AJJ Kindle G . Phenotype, genotype, treatment, and survival outcomes in patients with X-linked inhibitor of apoptosis deficiency. J Allergy Clin Immunol. (2022) 150:456–66. doi: 10.1016/j.jaci.2021.10.037 34920033

[121] Pachlopnik Schmid J Canioni D Moshous D Touzot F Mahlaoui N Hauck F . Clinical similarities and differences of patients with X-linked lymphoproliferative syndrome type 1 (XLP-1/SAP deficiency) versus type 2 (XLP-2/XIAP deficiency). Blood. (2011) 117:1522–9. doi: 10.1182/blood-2010-07-298372 21119115

[122] Gadoury-Levesque V Dong L Su R Chen J Zhang K Risma KA . Frequency and spectrum of disease-causing variants in 1892 patients with suspected genetic HLH disorders. Blood Adv. (2020) 4:2578–94. doi: 10.1182/bloodadvances.2020001605 PMC7322966 32542393

[123] Borte S von Dobeln U Hammarstrom L . Guidelines for newborn screening of primary immunodeficiency diseases. Curr Opin Hematol. (2013) 20:48–54. doi: 10.1097/MOH.0b013e32835a9130 23108220

[124] Lodi L Ricci S Romano F Ghiori F Canessa C Lippi F . Newborn screening for PIDs using both TREC and KREC identifies late occurrence of B cells. Pediatr Allergy Immunol. (2017) 28:498–500. doi: 10.1111/pai.12733 28517432

[125] Fazi C Lodi L Magi L Canessa C Giovannini M Pelosi C . Case report: Zellweger syndrome and humoral immunodeficiency: The relevance of newborn screening for primary immunodeficiency. Front Pediatr. (2022) 10:852943. doi: 10.3389/fped.2022.852943 35402347 PMC8990230

[126] Fischer A Provot J Jais JP Alcais A Mahlaoui N members of the CEREDIH French PID study group . Autoimmune and inflammatory manifestations occur frequently in patients with primary immunodeficiencies. J Allergy Clin Immunol. (2017) 140:1388–1393.e8. doi: 10.1016/J.JACI.2016.12.978 28192146

[127] Brisse E Wouters CH Andrei G Matthys P . How viruses contribute to the pathogenesis of hemophagocytic lymphohistiocytosis. Front Immunol. (2017) 8:1102. doi: 10.3389/fimmu.2017.01102 28936212 PMC5594061

[128] Amirifar P Ranjouri MR Abolhassani H Moeini Shad T Almasi-Hashiani A Azizi G . Clinical, immunological and genetic findings in patients with UNC13D deficiency (FHL3): A systematic review. Pediatr Allergy Immunol. (2021) 32:186–97. doi: 10.1111/PAI.13323 32679608

[129] Horne AC Wickstrom R Jordan MB Yeh EA Naqvi A Henter JI . How to treat involvement of the central nervous system in hemophagocytic lymphohistiocytosis? Curr Treat options neurology. (2017) 19(1):3. doi: 10.1007/S11940-017-0439-4 28155064 PMC5290057

[130] Henter JI Nennesmo I . Neuropathologic findings and neurologic symptoms in twenty-three children with hemophagocytic lymphohistiocytosis. J pediatrics. (1997) 130:358–65. doi: 10.1016/S0022-3476(97)70196-3 9063409

[131] Jordan MB Allen CE Greenberg J Henry M Hermiston ML Kumar A . Challenges in the diagnosis of hemophagocytic lymphohistiocytosis: Recommendations from the North American Consortium for Histiocytosis (NACHO). Pediatr Blood Cancer. (2019) 66:e27929. doi: 10.1002/pbc.27929 31339233 PMC7340087

