# Corrigendum: HLH as an additional warning sign of inborn errors of immunity beyond familial-HLH in children: a systematic review

**DOI:** 10.3389/fimmu.2024.1400034

**Published:** 2024-03-26

**Authors:** Silvia Ricci, Walter Maria Sarli, Lorenzo Lodi, Clementina Canessa, Francesca Lippi, Donata Dini, Marta Ferrari, Laura Pisano, Elena Sieni, Giuseppe Indolfi, Massimo Resti, Chiara Azzari

**Affiliations:** ^1^ Department of Health Sciences, University of Florence, Florence, Italy; ^2^ Immunology Division, Section of Pediatrics, Meyer Children’s Hospital IRCCS, Florence, Italy; ^3^ Department of Pediatrics, Meyer Children’s Hospital IRCCS, Florence, Italy; ^4^ Pediatric Hematology-Oncology Department, Meyer Children’s Hospital IRCCS, Florence, Italy; ^5^ Department Neurofarba, University of Florence, Florence, Italy

**Keywords:** hemophagocytic lymphohistiocytosis, inborn errors of immunity, macrophage activation syndrome, immune deficiency, familial hemophagocytic lymphohistiocytosis, hemophagocytic syndrome

In the published article, there was an error in affiliations 2, 3 and 4. Instead of “Meyer Childrens Hospital IRCCS”, it should be “Meyer Children’s Hospital IRCCS”.

The authors apologize for this error and state that this does not change the scientific conclusions of the article in any way. The original article has been updated.

In the published article, there was an error in the **Funding** statement. We have recently been informed by our institution that, for payment reimbursement purposes, due to a new Italian rule for Research Institutes, it is necessary to include the following acknowledgment in the published article: “This study was supported in part by funds from the ‘Current Research Annual Funding’ of the Italian Ministry of Health.” The correct **Funding** statement appears below.

**FUNDING**

“The author(s) declare that financial support was received for the research, authorship, and/or publication of this article. This study was supported in part by funds from the ‘Current Research Annual Funding’ of the Italian Ministry of Health.”

The authors apologize for this error and state that this does not change the scientific conclusions of the article in any way. The original article has been updated.

