# Supplementary material for: HLH as an additional warning sign of inborn errors of immunity beyond familial-HLH in children: a systematic review
Source: Front Immunol. 2024 Feb 13;15:1282804. doi: 10.3389/fimmu.2024.1282804 (PMC10896843; doi:10.3389/fimmu.2024.1282804)
Supplement: Supplementary file 1 [file DataSheet_1.docx]

SUPPLEMENTAL DATA

Sup 1. Search Strings

**PubMed**

("Primary Immunodeficiency Diseases"[MeSH Terms] OR ("Immunologic Deficiency Syndromes"[Mesh Terms] OR "inborn errors of immunity" [Title/Abstract] OR "congenital immunodeficiency" [Title/Abstract] OR "inherited immunodeficiency" [Title/Abstract] OR "immunodeficiency"[Title/Abstract] OR ("immunity"[Title/Abstract] AND ("defect"[Title/Abstract] OR "error"[Title/Abstract] OR "deficiency"[Title/Abstract])) OR "IEI"[Title/Abstract] OR "PID"[Title/Abstract] OR "Common Variable Immunodeficiency"[Mesh Terms] OR "Severe Combined Immunodeficiency"[Mesh Terms] OR "Ataxia Telangiectasia"[Title/Abstract] OR "Wiskott-Aldrich"[Title/Abstract] OR "Job Syndrome"[Title/Abstract] OR "ADA"[Title/Abstract] OR "hyper-igm"[Title/Abstract] OR " granulomatous disease, chronic"[Mesh Terms] OR "Combined Immunodeficiency "[Title/Abstract] OR "CVID"[Title/Abstract] OR "HIGM"[Title/Abstract] OR "SCID"[Title/Abstract] OR "CGD"[Title/Abstract] OR "XLA"[Title/Abstract] OR "HIES"[Title/Abstract] OR "ALPS"[Title/Abstract] OR "Hyper-IgE"[Title/Abstract] OR "SH2D1A"[Title/Abstract] OR "XIAP"[Title/Abstract] OR "NLRC4"[Title/Abstract] OR "CDC42"[Title/Abstract] OR "MAGT1"[Title/Abstract] OR "ITK"[Title/Abstract] OR "CD27"[Title/Abstract] OR "CD70"[Title/Abstract] OR "CTPS1"[Title/Abstract] OR "RASGRP1"[Title/Abstract] OR "SAP"[Title/Abstract] OR "X-linked lymphoproliferative syndrome"[Title/Abstract] OR ("lymphoproliferative"[Title/Abstract] NOT "leukemia"[Title/Abstract]) OR ("FAS"[Title/Abstract] NOT "fetal alcohol syndrome"[Title/Abstract]) OR "NEMO"[Title/Abstract] OR "22q11"[Title/Abstract] OR "Hereditary Autoinflammatory Diseases"[Mesh Terms]))

AND

("lymphohistiocytosis, hemophagocytic"[MeSH Terms] OR ("Macrophage Activation Syndrome"[MeSH Terms] OR "haemophag*"[Title/Abstract] OR "hemophag*"[Title/Abstract] OR "HLH"[Title/Abstract] OR (MAS [Title/Abstract] NOT ("motor assessment scale"[Title/Abstract] OR "meconium aspiration syndrome"[Title/Abstract])) OR "lymphohistiocyt*" [Title/Abstract] OR "histiocytic" [Title/Abstract]))

**Embase**

('hemophagocytic syndrome'/exp OR ('haemophag*' OR 'hemophag*' OR 'hlh' OR 'lymphohistiocyt*' OR (‘MAS’ NEAR/3 (‘macrophage’ OR ‘activation’)) OR 'macrophage activation syndrome' OR ‘hystiocitic'):ab,ti)

AND

('immune deficiency'/exp OR ('primary immunodeficiency diseases' OR 'immunologic deficiency syndromes' OR 'inborn errors of immunity' OR 'congenital immunodeficiency' OR 'inherited immunodeficiency' OR 'immunodeficiency' OR 'iei' OR 'pid' OR 'common variable immunodeficiency' OR 'severe combined immunodeficiency' OR 'ataxia telangiectasia' OR 'wiskott-aldrich' OR 'job syndrome' OR 'ada' OR 'hyper-igm' OR 'chronic granulomatous disease' OR 'combined immunodeficiency' OR 'cvid' OR 'higm' OR 'scid' OR 'cgd' OR 'xla' OR 'hies' OR 'alps' OR 'hyper-ige' OR 'sh2d1a' OR 'xiap' OR 'nlrc4' OR 'cdc42' OR 'magt1' OR 'itk' OR 'cd27' OR 'cd70' OR 'ctps1' OR 'rasgrp1' OR 'sap' OR 'x-linked lymphoproliferative syndrome' OR 'lymphoproliferative' OR 'fas' OR 'nemo' OR '22q11' OR 'hereditary autoinflammatory diseases' OR ('immunity' NEAR/3 ('defect' OR 'error' OR 'deficiency'))):ab,ti)

AND [embase]/lim NOT [medline]/lim

**Medline OVID**

(lymphohistiocytosis, hemophagocytic/ OR (macrophage activation syndrome OR hemophagocytic syndrome OR haemophag* OR hemophag* OR HLH OR lymphohistiocyt* OR (MAS ADJ3 (macrophage or activation)) OR hystiocitic).ab,ti.)

AND

(Primary immunodeficiency diseases/ OR (immune deficiency OR immunologic deficiency syndromes OR inborn errors of immunity OR congenital immunodeficiency OR inherited immunodeficiency OR immunodeficiency OR IEI OR PID OR common variable immunodeficiency OR severe combined immunodeficiency OR ataxia telangiectasia OR wiskott-aldrich OR job syndrome OR ADA OR hyper-igm OR chronic granulomatous disease OR combined immunodeficiency OR CVID OR HIGM OR SCID OR CGD OR XLA OR HIES OR ALPS OR HYPER-IGE OR SH2D1A OR XIAP OR NLRC4 OR CDC42 OR MAGT1 OR ITK OR CD27 OR CD70 OR CTPS1 OR RASGRP1 OR SAP OR x-linked lymphoproliferative syndrome OR lymphoproliferative OR fas OR nemo OR 22q11 OR hereditary autoinflammatory diseases OR (immunity ADJ3 (defect OR error OR deficiency))).ab,ti.)

**Cochrane Central**

'hemophagocytic syndrome' OR haemophag* OR hemophag* OR hlh OR lymphohistiocyt* OR (MAS NEAR/3 (macrophage OR activation)) OR 'macrophage activation syndrome' OR hystiocitic

AND

'immune deficiency' OR 'inborn errors of immunity' OR 'congenital immunodeficiency' OR 'inherited immunodeficiency' OR immunodeficiency OR iei OR pid OR 'common variable immunodeficiency' OR 'severe combined immunodeficiency' OR 'ataxia telangiectasia' OR wiskott-aldrich OR 'job syndrome' OR ada OR hyper-igm OR 'chronic granulomatous disease' OR 'combined immunodeficiency' OR cvid OR higm OR scid OR cgd OR xla OR hies OR alps OR hyper-ige OR sh2d1a OR xiap OR nlrc4 OR cdc42 OR magt1 OR itk OR cd27 OR cd70 OR ctps1 OR rasgrp1 OR sap OR 'x-linked lymphoproliferative syndrome' OR lymphoproliferative OR fas OR nemo OR 22q11 OR 'hereditary autoinflammatory diseases' OR (immunity NEAR/3 (defect OR error OR deficiency))

**Sup.2 Definitions**

**IEI**: 485 inherited disorders, often due to mutations in a single gene, involving specific impairment of normal development and immune function. IUIS2022 Classification groups IEI into 9 major categories and multiple subgroups based on which part of immune system is impaired (see Supplemental data for complete classification)^1^. IUIS2022 Classification also considers an additional group for phenocopies of IEI.

**FHL**: IEI which share HLH as their predominant clinical feature. According to IUIS 2022 Classification FHL can be distinguished in two subgroups based on the presence of hypopigmentation. To the first subgroup belong pathogenic mutations of PRF1, STX1, UNC13D, STXBP2 whereas the second subgroup comprises Chediak-Higashi syndrome, Griscelli type 2 and, rarely, Hermansky-Pudlak syndrome type 2.

**IEInotFHL**: IEI not belonging to FHL subgroups according to IUIS 2022 Classification.

**ESID Criteria**: criteria for clinical diagnosis of immunodeficiencies as defined by European Society for Immunodeficiency (ESID)^2^.

Sup.3 Emendment to protocol PROSPERO CRD42022371425.

The title has been updated.

Authors have been added.

No major changes from the original protocol.

Sup 4. Simplified IUIS 2022 Classification of IEI

| 1 Immunodeficiencies affecting cellular and humoral immunity (I) |
| --- |
| 1.1 T-B+ Severe Combined Immune Deficiency (SCID) |
| 1.2 T-B- Severe Combined Immune Deficiency (SCID) |
| 1.3 Combined Immunodeficiency (CID), Generally Less Profound than SCID |
| 2 Combined immunodeficiencies with associated or syndromic features (II) |
| 2.1 Immunodeficiency with Congenital Thrombocytopenia |
| 2.2 DNA Repair Defects Other Than Those Listed in Table 1 |
| 2.3 Thymic Defects with Additional Congenital Anomalies |
| 2.4 Immuno-osseous Dysplasias |
| 2.5 Hyper IgE Syndromes (HIES) |
| 2.6 Defects of Vitamin B12 and Folate Metabolism |
| 2.7 Anhidrotic Ectodermodysplasia with Immunodeficency (EDA-ID) |
| 2.8 Calcium Channel Defects |
| 2.9 Other Defects |
| 3 Predominantly antibody deficiencies (III) |
| 3.1 Severe Reduction in All Serum Immunoglobulin Isotypes with Profoundly Decreased or Absent B Cells, Agammaglobulinemia |
| 3.2 Severe Reduction in at Least 2 Serum Immunoglobulin Isotypes with Normal or Low Number of B Cells, CVID Phenotype |
| 3.3 Severe Reduction in Serum IgG and IgA with Normal/Elevated IgM and Normal Numbers of B cells, Hyper IgM |
| 3.4 Isotype, Light Chain, or Functional Deficiencies with Generally Normal Numbers of B Cells |
| 4 Diseases of immune dysregulation (IV) |
| 4.1 Familial Hemophagocytic Lymphohistiocytosis (FHL syndromes) |
| 4.2 Familial Hemophagocytic Lymphohistiocytosis (FHL syndromes) with hypopigmentation |
| 4.3 Regulatory T cell defects |
| 4.4 Autoimmunity with or without Lymphoproliferation |
| 4.5 Immune Dysregulation with Colitis |
| 4.6 Autoimmune Lymphoproliferative Syndrome (ALPS, Canale-Smith syndrome) |
| 4.7 Susceptibility to EBV and Lymphoproliferative Conditions |
| 5 Congenital defects of phagocyte number or function (V) |
| 5.1 Congenital Neutropenias |
| 5.2 Defects of Motility |
| 5.3 Defects of Respiratory Burst |
| 5.4 Other Non-Lymphoid Defects |
| 6 Defects in intrinsic and innate immunity (VI) |
| 6.1 Mendelian Susceptibility to mycobacterial disease (MSMD) |
| 6.2 Epidermodysplasia verruciformis (HPV) |
| 6.3 Predisposition to Severe Viral Infection |
| 6.4 Herpes Simplex Encephalitis (HSE) |
| 6.5 Predisposition to INVASIVE Fungal Diseases |
| 6.6 Predisposition to Mucocutaneous Candidiasis |
| 6.7 TLR Signaling Pathway Deficiency with Bacterial Susceptibility |
| 6.8 Other Inborn Errors of Immunity Related to Non-Hematopoietic Tissues |
| 6.9 Other Inborn Errors of Immunity Related to Leukocytes |
| 7 Autoinfammatory disorders (VII) |
| 7.1 Type 1 Interferonopathies |
| 7.2 Defects Affecting the Inflammasome |
| 7.3 Non-Inflammasome Related Conditions |
| 8 Complement defciencies (VIII) |
| 9 Bone marrow failure (IX) |
| 10 Phenocopies of inborn errors of immunity (X) |

Sup 5. List of Mutations

Sup 6. The Joanna Briggs Institute (JBI) Critical Appraisal Checklist for Case Reports

| Q1 | Were patient’s demographic characteristics clearly described? |
| --- | --- |
| Q2 | Was the patient’s history clearly described and presented as a timeline? |
| Q3 | Was the current clinical condition of the patient on presentation clearly described? |
| Q4 | Were diagnostic tests or assessment methods and the results clearly described? |
| Q5 | Was the intervention(s) or treatment procedure(s) clearly described? |
| Q6 | Was the post-intervention clinical condition clearly described? |
| Q7 | Were adverse events (harms) or unanticipated events identified and described? |
| Q8 | Does the case report provide takeaway lessons? |

|  | Yes |
| --- | --- |
|  | No |
|  | Unclear |
|  | Not Applicable |

| Study | Year | Q1 | Q2 | Q3 | Q4 | Q5 | Q6 | Q7 | Q8 |
| --- | --- | --- | --- | --- | --- | --- | --- | --- | --- |
| Agarwal A | 2016 |  |  |  |  |  |  |  |  |
| Al-Hammadi S | 2021 |  |  |  |  |  |  |  |  |
| Alsalamah M | 2017 |  |  |  |  |  |  |  |  |
| Alsalamah M | 2015 |  |  |  |  |  |  |  |  |
| Aricò M | 1999 |  |  |  |  |  |  |  |  |
| Aytekin ES | 2021 |  |  |  |  |  |  |  |  |
| Bajaj P | 2014 |  |  |  |  |  |  |  |  |
| Barsalou J | 2018 |  |  |  |  |  |  |  |  |
| Bird JA | 2009 |  |  |  |  |  |  |  |  |
| Boehmer DFR | 2020 |  |  |  |  |  |  |  |  |
| Burak N | 2021 |  |  |  |  |  |  |  |  |
| Burns C | 2016 |  |  |  |  |  |  |  |  |
| Butt FF | 2022 |  |  |  |  |  |  |  |  |
| Cesaro S | 2003 |  |  |  |  |  |  |  |  |
| Celiksoy MH | 2018 |  |  |  |  |  |  |  |  |
| Chidambaram AC | 2020 |  |  |  |  |  |  |  |  |
| Cui T | 2020 |  |  |  |  |  |  |  |  |
| De la Varga-Martínez R | 2017 |  |  |  |  |  |  |  |  |
| Dvorak CC | 2008 |  |  |  |  |  |  |  |  |
| Eng V | 2020 |  |  |  |  |  |  |  |  |
| Escaron C | 2022 |  |  |  |  |  |  |  |  |
| Gothe F | 2022 |  |  |  |  |  |  |  |  |
| Greil J | 2016 |  |  |  |  |  |  |  |  |
| Grunebaum E | 2000 |  |  |  |  |  |  |  |  |
| Halasa NB | 2003 |  |  |  |  |  |  |  |  |
| Han SP | 2019 |  |  |  |  |  |  |  |  |
| Harnisch E | 2016 |  |  |  |  |  |  |  |  |
| Hügle B | 2006 |  |  |  |  |  |  |  |  |
| Higuchi T | 2022 |  |  |  |  |  |  |  |  |
| Hoshino T | 2005 |  |  |  |  |  |  |  |  |
| Honda K | 2000 |  |  |  |  |  |  |  |  |
| Horneff G | 2013 |  |  |  |  |  |  |  |  |
| Imashuku S | 2002 |  |  |  |  |  |  |  |  |
| Jain G | 2022 |  |  |  |  |  |  |  |  |
| Jiang MY | 2016 |  |  |  |  |  |  |  |  |
| Klemann C | 2017 |  |  |  |  |  |  |  |  |
| Kuijpers TW | 2011 |  |  |  |  |  |  |  |  |
| Lekbua A | 2019 |  |  |  |  |  |  |  |  |
| Liang J | 2017 |  |  |  |  |  |  |  |  |
| Liang J | 2019 |  |  |  |  |  |  |  |  |
| Loganathan A | 2020 |  |  |  |  |  |  |  |  |
| Lougaris V | 2020 |  |  |  |  |  |  |  |  |
| Maignan M | 2013 |  |  |  |  |  |  |  |  |
| Malkan UY | 2015 |  |  |  |  |  |  |  |  |
| Mischler M | 2007 |  |  |  |  |  |  |  |  |
| Ozturk C | 2013 |  |  |  |  |  |  |  |  |
| Pasic S | 2012 |  |  |  |  |  |  |  |  |
| Pasic S | 2003 |  |  |  |  |  |  |  |  |
| Pachlopnik Schmid JM | 2006 |  |  |  |  |  |  |  |  |
| Patiroglu T | 2014 |  |  |  |  |  |  |  |  |
| Prader S | 2021 |  |  |  |  |  |  |  |  |
| Qiu KY | 2017 |  |  |  |  |  |  |  |  |
| Razaghian A | 2020 |  |  |  |  |  |  |  |  |
| Ren Y | 2021 |  |  |  |  |  |  |  |  |
| Ricci S | 2017 |  |  |  |  |  |  |  |  |
| Rossi-Semerano L | 2011 |  |  |  |  |  |  |  |  |
| Rudman Spergel A | 2013 |  |  |  |  |  |  |  |  |
| Scheffler-Mendoza SC | 2014 |  |  |  |  |  |  |  |  |
| Schmid I | 2002 |  |  |  |  |  |  |  |  |
| Seidel MG | 2012 |  |  |  |  |  |  |  |  |
| Shi B | 2020 |  |  |  |  |  |  |  |  |
| Sirinavin S | 2004 |  |  |  |  |  |  |  |  |
| Spinner MA | 2002 |  |  |  |  |  |  |  |  |
| Staines-Boone AT | 2017 |  |  |  |  |  |  |  |  |
| Szczawinska-Poplonyk A | 2020 |  |  |  |  |  |  |  |  |
| Triebwasser MP | 2021 |  |  |  |  |  |  |  |  |
| Tucci F | 2021 |  |  |  |  |  |  |  |  |
| Uslu N | 2009 |  |  |  |  |  |  |  |  |
| Valentine G | 2014 |  |  |  |  |  |  |  |  |
| Van Montfrans JM | 2009 |  |  |  |  |  |  |  |  |
| Vieth S | 2013 |  |  |  |  |  |  |  |  |
| Voeten M | 2014 |  |  |  |  |  |  |  |  |
| Wegehaupt O | 2020 |  |  |  |  |  |  |  |  |
| Wei A | 2020 |  |  |  |  |  |  |  |  |
| White S | 2019 |  |  |  |  |  |  |  |  |
| Zhou S | 2017 |  |  |  |  |  |  |  |  |
| Zhou Z | 2021 |  |  |  |  |  |  |  |  |

Sup 7. The Joanna Briggs Institute (JBI) Critical Appraisal Checklist for Case Series

| Q1 | Were there clear criteria for inclusion in the case  series? |
| --- | --- |
| Q2 | Was the condition measured in a standard, reliable  way for all participants included in the case series? |
| Q3 | Were valid methods used for identification of the  condition for all participants included in the case  series? |
| Q4 | Did the case series have consecutive inclusion of  participants? |
| Q5 | Did the case series have complete inclusion of  participants? |
| Q6 | Was there clear reporting of the demographics of  the participants in the study? |
| Q7 | Was there clear reporting of clinical information of  the participants? |
| Q8 | Were the outcomes or follow up results of cases  clearly reported? |
| Q9 | Was there clear reporting of the presenting  site(s)/clinic(s) demographic information? |
| Q10 | Was statistical analysis appropriate? |

|  | Yes |
| --- | --- |
|  | No |
|  | Unclear |
|  | Not Applicable |

| Author | Year | Q1 | Q2 | Q3 | Q4 | Q5 | Q6 | Q7 | Q8 | Q9 | Q10 |
| --- | --- | --- | --- | --- | --- | --- | --- | --- | --- | --- | --- |
| Alawbathani S | 2022 |  |  |  |  |  |  |  |  |  |  |
| Bode SFN | 2015 |  |  |  |  |  |  |  |  |  |  |
| Castro CN | 2020 |  |  |  |  |  |  |  |  |  |  |
| Cetinkaya PG | 2020 |  |  |  |  |  |  |  |  |  |  |
| Gera A | 2021 |  |  |  |  |  |  |  |  |  |  |
| Kashiwagi Y | 2007 |  |  |  |  |  |  |  |  |  |  |
| Lam MT | 2020 |  |  |  |  |  |  |  |  |  |  |
| Le Voyer T | 2021 |  |  |  |  |  |  |  |  |  |  |
| Marsh RA | 2010 |  |  |  |  |  |  |  |  |  |  |
| Martin A | 2009 |  |  |  |  |  |  |  |  |  |  |
| Marzollo A | 2022 |  |  |  |  |  |  |  |  |  |  |
| Parekh C | 2011 |  |  |  |  |  |  |  |  |  |  |
| Prader S | 2018 |  |  |  |  |  |  |  |  |  |  |
| Salzer E | 2013 |  |  |  |  |  |  |  |  |  |  |
| Schaballie H | 2013 |  |  |  |  |  |  |  |  |  |  |
| Schultz KA | 2008 |  |  |  |  |  |  |  |  |  |  |
| Shadur B | 2019 |  |  |  |  |  |  |  |  |  |  |
| Sheth J | 2019 |  |  |  |  |  |  |  |  |  |  |
| Sieni E | 2012 |  |  |  |  |  |  |  |  |  |  |
| Squire JD | 2020 |  |  |  |  |  |  |  |  |  |  |
| Stepensky P | 2011 |  |  |  |  |  |  |  |  |  |  |
| Suzuki N | 2009 |  |  |  |  |  |  |  |  |  |  |
| Tesi B | 2015 |  |  |  |  |  |  |  |  |  |  |
| Vavassori S | 2021 |  |  |  |  |  |  |  |  |  |  |
| Vignesh P | 2022 |  |  |  |  |  |  |  |  |  |  |
| Yang X | 2015 |  |  |  |  |  |  |  |  |  |  |
| Yang X | 2012 |  |  |  |  |  |  |  |  |  |  |
| Yao J | 2022 |  |  |  |  |  |  |  |  |  |  |
| Zhang R | 2018 |  |  |  |  |  |  |  |  |  |  |
| Zheng F | 2016 |  |  |  |  |  |  |  |  |  |  |

Sup 8. List of IEInotfHL identified according to IUIS 2022 classification

| IEI identified with molecular test | | |
| --- | --- | --- |
| Group, subgroup of IEI and IEI | | Patients |
| **immunodeficiencies affecting cellular and humoral immunity (i)** | |  |
| *T-B+ Severe Combined Immune Deficiency (SCID)* | γc deficiency (IL2RG) | 10 |
|  | cd3δ deficiency | 1 |
|  | Cd3ε deficiency | 1 |
|  | IL7Rα deficiency | 1 |
| *T-B-SCID* | RAG1 deficiency | 4 |
|  | ADA SCID | 2 |
| *Combined Immunodeficiency (CID), Generally Less Profound than SCID* | ITK deficiency | 6 |
|  | HELIOS deficiency (IKZF2) | 2 |
|  | CD40L deficiency | 1 |
|  | DOCK2 deficiency | 1 |
|  | ZAP70 deficiency | 1 |
| **Combined immunodeficiencies with associated or syndromic features (II)** | |  |
| *Thymic Defects with Additional Congenital Anomalies* | 22q11.2 Deletion Syndrome | 4 |
| *Immunodeficiency with Congenital Thrombocytopenia* | Wiskott-Aldrich Syndrome | 3 |
| *Hyper IgE Syndromes (HIES)* | AD-HIES STAT3 deficiency | 2 |
| *DNA Repair Defects Other* | Ataxia Teleangiectasia | 2 |
|  | ICF type 2 | 1 |
| *Anhidrotic Ectodermodysplasia with ID (EDA-ID)* | NEMO /IKBKG deficiency | 2 |
| *Calcium Channel defects* | ORAI1 | 1 |
| **Predominantly antibody deficiencies (III)** | |  |
| *Severe Reduction in All Serum Immunoglobulin Isotypes with Profoundly Decreased or Absent B Cells, Agammaglobulinemia* | BTK defiency (XLA) | 4 |
| *Severe Reduction in at Least 2 Serum Immunoglobulin Isotypes with Normal or Low Number of B Cells, CVID Phenotype* | APDS1 (PIK3CD) | 3 |
| **Disease of immune dysregulation (IV)** | |  |
| *Susceptibility to EBV and Lymphoproliferative Conditions* | SAP deficiency (XLP1) | 16 |
|  | XIAP deficiency (XLP2) | 14 |
|  | CD27 deficiency | 3 |
| *Regulatory T Cell Defects* | LRBA deficiency | 2 |
| *Autoimmune Lymphoproliferative Syndrome (ALPS, Canale-Smith syndrome)* | ALPS (FAS) | 1 |
|  | ALPS (TNFRFS6) | 1 |
| **Congenital defects of phagocyte number or function (V)** | |  |
| *Defects of Respiratory Burst* | XL-CGD | 14 |
|  | AR CGD (NCF1) | 4 |
|  | AR CGD (NCF2) | 1 |
|  | AR-CGD (CYBA) | 1 |
| *Other Non-Lymphoid Defects* | GATA2 deficiency | 3 |
| *Congenital Neutropenias* | Shwachman-Diamond (SBDS) | 1 |
| **Defects in intrinsic and innate immunity (VI)** | |  |
| *Predisposition to Severe Viral Infection* | ZNFX1 deficiency | 9 |
|  | STAT1 deficiency | 5 |
|  | IFNαR1 deficiency | 1 |
| *MSMD* | IFNγR1 deficiency | 4 |
|  | gp91phox deficiency (CYBB) ^19^ | 1 |
| *Predisposition to Mucocutaneous Candidiasis* | STAT 1 GoF | 2 |
| *Other Inborn Errors of Immunity Related to Non-Hematopoietic Tissues* | HMOX1 deficiency | 1 |
| **Autoinflammatory disorders (VII)** | |  |
| *Defects Affecting the Inflammasome* | FMF | 2 |
|  | NCLR4 GoF | 2 |
| *Non-Inflammasome Related Conditions* | NCKAP1L deficiency | 2 |
|  | TIM3 deficiency (HAVCR2) | 1 |
|  | TRAPS (TNFRSF1A) | 1 |
| *Type 1 Interferonopathies* | CDC42 deficiency | 4 |
| **Bone Marrow Failure (IX)** | |  |
| *Bone Marrow Failure* | DKCX1 deficiency | 1 |
| IEI identified clinically or through functional test | | |
| Group, subgroup of IEI and IEI | | Patients |
| **Immunodeficiencies affecting cellular and humoral immunity (I)** | |  |
| *T-B+ Severe Combined Immune Deficiency (SCID)* |  | 1 |
| *T-B-SCID* |  | 1 |
| *SCID not classified* |  | 3 |
| *Combined Immunodeficiency (CID), Generally Less Profound than SCID* |  | 10 |
| **Combined immunodeficiencies with associated or syndromic features (II)** | |  |
| *DNA Repair Defects Other* | Ataxia Teleangiectasia | 1 |
| **Predominantly antibody deficiencies (III)** | |  |
| *Severe Reduction in at Least 2 Serum Immunoglobulin Isotypes with Normal or Low Number of B Cells, CVID Phenotype* | CVID | 3 |
| *Isotype, Light Chain, or Functional Deficiencies with Generally Normal Numbers of B Cells* | SIgM deficiency | 1 |
| **Disease of immune dysregulation (IV)** | |  |
| *Susceptibility to EBV and Lymphoproliferative Conditions* | SAP deficiency (XLP1) | 2 |
| **Congenital defects of phagocyte number or function (V)** | |  |
| *Defects of Respiratory Burst* | CGD | 6 |
| **Defects in intrinsic and innate immunity (VI)** | |  |
| *Epidermodysplasia verruciformis (HPV)* | WHIM | 1 |

Sup 9. List of Triggers identified according to Group of IEI (IUIS 2022)

| Group of IEI and Trigger for HLH | Patients |
| --- | --- |
| **Immunodeficiencies affecting cellular and humoral immunity- SCID and CID (I)** |  |
| EBV | 12 |
| CMV | 9 |
| Adenovirus | 4 |
| *K.pneumoniae* | 3 |
| BCG | 3 |
| H1N1 | 1 |
| *S.maltophilia* | 1 |
| Rotavirus/norovirus | 1 |
| Alternaria | 1 |
| Aspergillus | 1 |
| *P.aeuruginosa* | 1 |
| *P. jirovecii* | 1 |
| *M. tuberculosis* | 1 |
| Multiple triggers | 5 |
| Not identified | 10 |
| **Combined immunodeficiencies with associated or syndromic features (II)** |  |
| EBV^8^ | 6 |
| CMV | 4 |
| *P.jirovecii* | 2 |
| *K.pneumoniae* | 1 |
| Parvovirus | 1 |
| Multiple triggers | 3 |
| Not identified | 5 |
| **Predominantly antibody deficiencies (III)** |  |
| EBV | 4 |
| Adenovirus | 2 |
| Not identified | 4 |
| **Disease of immune dysregulation (IV)** |  |
| EBV | 19 |
| CMV | 2 |
| Parvovirus | 1 |
| HHV8 | 1 |
| SARS-CoV2 | 1 |
| Multiple triggers | 1 |
| Not identified | 16 |
| **Congenital defects of phagocyte number or function (V)** |  |
| *Burkholderia spp*. (cepacia or multivorans) | 7 |
| Leishmania | 4 |
| *Candida spp*. (lusitania or parapsilosis) | 3 |
| *Staphilococcus spp*. (aureus or hominis) | 3 |
| EBV | 3 |
| CMV | 2 |
| *Salmonella spp.* | *2* |
| HSV1 | 1 |
| VZV | 1 |
| Penicillium | 1 |
| *Pseudomonas spp.* | 1 |
| *E.cloacae* | 1 |
| Multiple triggers | 6 |
| Not identified | 5 |
| **Defects in intrinsic and innate immunity (VI)** |  |
| BCG | 5 |
| EBV | 3 |
| CMV | 2 |
| HHV6 | 3 |
| Live strain viral vaccines (MMR, varicella) | 3 |
| *Staphylococcus spp*. | 2 |
| Rotavirus, norovirus, sapovirus | 2 |
| Adenovirus | 1 |
| Parainfluenza virus | 1 |
| *M.tuberculosis* | 1 |
| Multiple triggers | 3 |
| Not identified | 6 |
| **Autoinflammatory disorders (VII)** |  |
| BCG | 1 |
| **Bone Marrow Failure (IX)** |  |
| EBV | 1 |

Sup 10. Laboratory features (median and IQR) for groups of IEI according with IUIS 2022 Classification

|  | I SCID and CID | II Syndromic CID | III Antibody deficiencies | IV Immune dysregulation |
| --- | --- | --- | --- | --- |
| Ferritin µg/L | 5866 (2461 – 18351) | 4551.5 (2393 – 33187.5) | 15125 (1955.25 – 50799.25) | 4804 (1643 – 10912) |
| sIL2R U/mL | 1857.2 (1278.25 – 3745.25) | 6671.5 (4362 – 9327.5) | 4972 | 4780 (1691.5 – 8931.05) |
| Fibrinogen mg/dL | 110 (88.5 – 167) | 140 (93 – 146) | 125 (91.1 – 156.5) | 147.5 (120.25 – 176.5) |
| Triglycerides mg/dL | 386 (317.75 – 540.25) | 350.5 (319 – 666.75) | 388 (311-511.175) | 315.5 (281.5 -360.5) |
| Hemoglobin g/dL | 7.65 (6.8 – 7.93) | 7.6 (6.7 – 8.3) | 8 (6.7 – 8.7) | 8.9 (8.1 – 9.27) |
| Neutrophils /µL | 500 (185 – 1857) | 1150 (885 – 2140) | 660 (420 – 3840) | 1220 (302 – 2282) |
| Platelets /µL | 38000 (18750 – 61000) | 38000 (25250 – 51000) | 24000 (14000 – 47000) | 51000 (19750 – 92000) |
| ALT (U/L) | 115.5 (79.25 – 371.25) | 95.5 (71.25 – 119.75) | 98 (79.5 – 421) | 277.1 (108 – 453) |
| AST (U/L) | 264.5 (149.75 – 1312.5) | 198 (162.5 – 233.5) | 165.5 (94 – 1017.5) | 289 (133.25 – 546.75) |
| Serum IgG (mg/dL) | 260 (160 – 463) | 192 (152.75 -862.75) | 641 (535 – 1125.5) | 641 (451 – 1100) |
| Serum IgA (mg/dL) | 51.85 (7.62 – 98.25) | 136 (49.25 – 169.5) | 99 (76 – 135) | 111 (55 – 302) |
| Serum IgM (mg/dL) | 26 (17.05 – 36.45) | 156 (85.75 – 605) | 43 (18.3 – 89.5) | 76 (36.5 – 662.75) |
| CD3+ | 17 (6 – 32) | 7282 (4518 – 1679) | 4787 (3973 – 5601) | 1393 (969 – 1607) |
| CD3+CD4+ | 16 (6 – 22) | 9679 (8024 – 11334) | 429 (243 – 615) | 984 (469 – 1420) |
| CD3+CD8+ | 1 (0 – 8) | 6765 (3823 – 9707) | 4434 (3762 – 5106) | 680 (467 – 1000) |

|  | V Phagocyte defects | VI Innate immunity defects | VII Autoinflammatory defects |
| --- | --- | --- | --- |
| Ferritin µg/L | 2500 (1281 – 10356.5) | 4890 (2800 – 22935) | 8964.7 (1957.75 – 23033.25) |
| sIL2R U/mL | 11900 (3456.5 – 16955.5) | 6933 (4074.5 – 13341) | 3459 (2854 – 6370.25) |
| Fibrinogen mg/dL | 110 (91.25 – 146) | 90 (79 – 170) | 130 (101 – 262) |
| Triglycerides mg/dL | 310 (266 – 438) | 351.5 (271.25 – 708.25) | 434 (349.75 – 626.25) |
| Hemoglobin g/dL | 6.4 (5.8 – 7.8) | 7.1 (6.7 – 7.6) | 8.9 (6.6 – 9.5) |
| Neutrophils /µL | 1372 (810 – 1923) | 5800 (2450 – 13065) | 475 (92 – 1442) |
| Platelets /µL | 40000 (19500 -80000) | 23500 (16250 – 66000) | 54000 (37000 – 81000) |
| ALT (U/L) | 307 (113 – 606) | 834.5 (558.5 – 982.25) | 403 (296.5 – 1507.5) |
| AST (U/L) | 490 (205 – 1670) | 2886.5 (1015.75 – 3115.5) | 525 (379.5 – 1260) |
| Serum IgG (mg/dL) | 650 (406.5 -825) | 401.5 (400.75 - 402.25) | 2581 (1530 – 9240.5) |
| Serum IgA (mg/dL) | 89.35 (57.52 -121.17) | 49 (46 – 52) | 975 (552.5 – 1397.5) |
| Serum IgM (mg/dL) | 178.75 (112.62 -244.87) | 210.5 (146.25 – 274.75) | 675.5 (343.25 – 1007.75) |
| CD3+ | 740 (553 – 926) | - | 930 (680 – 20865) |
| CD3+CD4+ | 574 (326 – 1285) | 5080 | 250 (222.5 – 5225) |
| CD3+CD8+ | 482 (389 – 1068) | 1670 | 677 (418.5 – 15638) |

Sup 11. Previous clinical manifestations of patients with HLH before IEI diagnosis

| Clinical preceding signs | Patients |
| --- | --- |
| Infections  Multiple-site infections | 38 (29.9%)  15 (11.8%) |
| Pneumonia  Recurrent pneumonias  Fungal pneumonia | 17 (13.4%)  10/17 (58.9%)  1/17 (5.9%) |
| Upper Respiratory Tract Infections | 11 (8.7%) |
| Recurrent OMA | 7 (5.5%) |
| Chronic/recurrent sinusitis | 4 (3.1%) |
| Sepsis | 4 (3.1%) |
| Candidiasis (cutaneous or mucosal) | 4 (3.1%) |
| Skin, perianal, visceral abscesses | 3 (2.3%) |
| Lymphadenitis | 2 (1.6%) |
| Severe skin infections | 2 (1.6%) |
| Live-strain-vaccine infections (polio, VZV) | 2 (1.6%) |
| Osteomyelitis | 1 (0,8%) |
| Meningoencephalitis | 1 (0,8%) |
| Other | 4 (3.1%) |
| Cytopenia, splenomegaly or other hematologic anomalies | 13 (10.2%) |
| Failure to Thrive | 10 (7.9%) |
| Skin anomalies or rash | 5 (3.9%) |
| Facial dysmorphisms | 5 (3.9%) |
| Chronic Diarrhea  Highly suspected VEO-IBD | 8 (6.3%)  1 (0.8%) |
| Tumors | 1 (0.8%) |
| Developmental delay | 5 (3.9%) |
| Recurrent Fevers | 4 (3.1%) |
| Cardiac anomalies | 2 (1.6%) |
| Other (arthritis/arthralgias, liver disfunction, bone anomalies, weight loss…) | 17 (13.4%) |

Sup 12. Synthesis of included reports.

| AUTHOR | DESIGN | SETTING, COUNTRY | POPULATION | OBJECTIVE | RESULT |
| --- | --- | --- | --- | --- | --- |
| Agarwal A | Case Report | Narayana Multispecialty Hospital, Jaipur, India | 1 patient with selective IgM deficiency | To describe a case of HLH in a patient with SIGM deficiency | A 27-year-old boy with a recent history of severe bilateral pneumonia that required admission to the intensive care unit and then was re-hospitalized for sepsis developed HLH but no trigger was found. Investigation revealed profound selective IgM deficiency with total absence of CD19 B lymphocytes in bone marrow immunophenotyping. A diagnosis of HLH with selective IgM deficiency was made. |
| Alsalamah M | Case Report | The Hospital for Sick Children, Toronto, Canada | 1 patient with Ataxia Teleangiectasia | To describe a case of HLH in a patient with Ataxia Telangiectasia | A child of Italian origin born to consanguineous parents had a clinical and laboratory diagnosis of Ataxia Telangiectasia at age 6 years for a history of developmental delay, ataxia, dysarthria, and ocular telangiectasias. At 11 years of age, he developed HLH. No underlying cause was found. Immediately after recognition of HLH, antibiotic therapy was replaced with dexamethasone, but he died after 2 days of therapy. |
| Alsalamah M | Case Report | The Hospital for Sick Children, Toronto, Canada | 1 patient with CD3delta deficiency | To describe a case of HLH in a patient with CD3delta deficiency | A Mennonite woman with a medical history of eczema, mouth ulcers, and refractory oral and diaper candidiasis presented at age 6 months with vomiting, diarrhea, and lethargy. The patient developed treatment refractory HLH leading to multi-organ failure. Immunologic evaluation was diagnostic for SCID, and postmortem genetic testing confirmed a homozygous mutation in CD38, previously described in Mennonites. First case of HLH in CD3delta deficiency. |
| Alawbathani S | Case Series | Multiple Hospitals, CENTOGENE GmbH, Rostock, Germany | 9 patients with ZNFX1 variants | To describe clinical and molecular features of ZNFX1 variants | 9 patients from different families around the world were identified through a comprehensive global database of genotypes and phenotypes for rare diseases. 3 of the 9 patients from the Middle East and Turkey developed HLH-like episodes in the first few months of life. One of them, with a novel VUS variant, presented with septic shock caused by *S.aureus* infection complicated by HLH at the age of 3 months. She died at 12 months of age following recurrence of severe bacterial infection complicated by episodes of HLH. |
| Al-Hammadi S | Case Report | Tawam Hospital, Al Ain, Abu Dhabi, United Arab Emirates | 1 patient with Chronic Granulomatous Disease | To provide further justifications for establishing safe rules for BCG vaccination | A 3-month-old infant with X-linked MSMD (CYBB) developed HLH and multiorgan failure after BCG vaccine. He did not receive treatment for BCG. He died after 5 days of intravenous steroid therapy. |
| Aricò M | Case Report | Clinica Pediatrica, IRCCS Policlinico S. Matteo, Pavia, Italy | 1 patient with 22Q11.2 Deletion Syndrome | To describe a case of HLH in a patient with 22q11.2 Deletion Syndrome | A 5.3-year-old female with a history of tetralogy of Fallot was admitted for recurrent muco-cutaneous hemorrhage. Poor growth and dysmorphic facial features were noted on admission. The laboratory showed hypoparathyroidism and bilinear cytopenia, and bone marrow aspirate showed erythrophagocytosis. Intravenous steroid and immunoglobulin therapy was started. FISH confirmed the diagnosis of 22q11.2 deletion syndrome. After the first episode, the patient developed 2 more episodes of hyperinflammation in 3 months. The diagnosis of HLH was made and treatment with HLH94 was started, but the patient died at 6.1 years. |
| Aytekin ES | Case Report | Hacettepe University Faculty of Medicine, Ankara, Turkey | 1 patient with DOCK2 deficiency | To describe a case of HLH after HSCT in a patient with DOCK2 deficiency | A 12-month-old child was admitted to the hospital for recurrent diarrhea and poor growth. On examination, profound CD4 T naive lymphopenia was found. Shortly thereafter, a molecular diagnosis of DOCK2 deficiency SCID was made, and an HSCT was performed at 14 months. 3 months after HSCT, the patients developed EBV-triggered HLH. At that time, the chimerism was 6%. Treatment was started according to HLH 04 protocol, but the patient died of ARDS before a new HSCT was performed. |
| Bajaj P | Case Report | Section of Allergy and Immunology, The Pennsylvania State University Milton S. Hershey Medical Center, Hershey, Pennsylvania, USA | 1 patient with CVID | To describe a case of HLH in a patient with CVID | A 52-year-old man with a history of CVID diagnosed 8 years earlier was hospitalized for persistent high-grade fever for 3 weeks. After admission, he developed severe pancytopenia with shock and MOF. Peripheral blood smear showed marked anisocytosis and poikilocytosis with elevated atypical lymphocytes. Flow cytometry showed a markedly elevated CD8 count with an abnormal CD4/CD8 ratio. Monospot test was negative, but real-time polymerase chain reaction showed elevated EBV load. The patient was treated according to HLH04 protocol for his HLH, but his clinical condition rapidly deteriorated and he died from MOF. |
| Barsalou J | Case Report | CHU Sainte Justine, Montréal, QC, Canada | 1 patient with NLRC4 GOF | To propose the benefit of rapamycin for NLRC4 associated MAS | A 12-day-old child was hospitalized for severe diarrhea, weight loss, acidosis, and vasoplegic shock. Further evaluation showed criteria for MAS, so therapy with intravenous methylprednisolone, anakinra and rapamycin was started, with clinical remission. Subsequently, a de novo heterozygous mutation in the nucleotide-binding domain of NLRC4 was found, and functional testing revealed self-activation of the protein leading to MAS. |
| Bird JA | Case Report | Baylor College of Medicine and Texas Children’s Hospital, | 1 patient with SH2D1A mutation | To describe a case of HLH in a patient with X linked lymphoproliferative syndrome | A 24-month-old child with familiarity for XLP was first evaluated for persistent fever and pneumonia. The culture was positive for Adenovirus. After 6 weeks, he was hospitalized for persistent fever, neurological signs, and diffuse maculo-papular rash. Further laboratory and instrumental investigations, in addition to clinical signs, were consistent with HLH. Treatment with dexamethasone, cyclosporine and etoposide was started. PCR for parvovirus was positive. The patient later developed recurrent HLH and died of multi-organ failure due to Enterobacter infection. Molecular analysis confirmed the mutation of SH2D1A. |
| Bode SFN | Case Series | Multiple Hospitals | 28 patients with confirmed or highly suspected IEI | To describe clinical and laboratory findings in patients with IEI and HLH | This study identified 63 patients with IEI other than genetic disorders of cytotoxicity or XLP, who met the current clinical criteria for HLH. Twenty-eight patients with confirmed or highly suspected IEI were enrolled in the study through a multicenter survey. Another 35 patients were enrolled from the literature. In 36 of them, HLH syndrome was the initial manifestation before/at the diagnosis of IEI. Compared with patients with cytotoxicity defects, patients with T-cell deficiency had lower levels of soluble CD25 and higher concentrations of ferritin. HLH also appeared in patients with severe T-cell and NK-cell deficiencies. The authors pointed out that current criteria for HLH diagnosis are unable to differentiate HLH with different pathogenesis. |
| Bohemer DFR | Case Report | University Hospital, Ludwig-Maximilians-Universität München, Munich, Germany | 1 patient with STAT1 LOF | To characterize a novel STAT1 loss-of-function variant | A 13-month-old child born to consanguineous parents presented with multiple severe viral infections that eventually triggered HLH and liver failure. Exome sequencing detected a novel homozygous STAT1 variant. Functional testing revealed that this variant leads to loss of STAT1 protein expression with susceptibility to viral infections and hyperinflammation. The child underwent HSCT but died 64 days later due to CMV reactivation and severe acute respiratory distress syndrome. |
| Burak N | Case Report | Morristown Medical Center, Morristown, NJ, USA | 1 patient with GATA2 deficiency | To describe a case of HLH in a patient with GATA2 deficiency | A 22-year-old woman with a history of congenital right kidney absence, hearing loss, and leukopenia presented with a 3-week history of generalized malaise, fever, chest pain, cough, and shortness of breath. She developed acute systemic CMV infection, further complicated by HLH. An immunodeficiency genetic panel revealed a monoallelic mutation in GATA2, a gene encoding zinc transcription factors responsible for regulating hematopoiesis. The patient was treated with the HLH94 protocol and improved rapidly without the need for etoposide. She then underwent HSCT approximately 11 months after diagnosis. |
| Burns C | Case Report | The Children’s Cancer Centre, The Royal Children’s Hospital, Melbourne, Australia | 1 patient with STAT1 LOF | To describe a case of HLH in a patient with STAT1 deficiency | An 8-month-old child was hospitalized for 5 days of irritability, fever, and a diffuse, scalding rash without mucosal involvement. Further evaluation was consistent with an HHV6-induced HLH. He was treated with IVIG and antibiotics with resolution. At 13 months, he developed a new outbreak of HLH after MMR vaccination. He later became encephalopathic and required intensive care support. He was treated with dexamethasone. Symptoms regressed in 2 weeks without change of treatment. He then underwent HSCT with complete resolution. WES identified a novel homozygous mutation of STAT1. Functional testing confirmed hypo-phosphorylation of STAT1. |
| Butt FF | Case Report | Pediatric Hematology and Oncology Department, Dubai Hospital, Dubai, United Arab Emirates | 1 patient with Chronic Granulomatous Disease | To describe a case of HLH in a patient with NCF2 mutation | A 3-month-old girl was admitted because of severe recurrent infections and persistent fever. She had a history of frequent hospitalizations for infections. Bronchoalveolar lavage culture showed the growth of *Pseudomonas aeruginosa*. A broad spectrum of antibiotics and antifungals were administered during her hospitalization. Blood culture showed positivity for *S.hominis*. Further examination associated with clinical signs raised suspicion of HLH. A homozygous pathogenic variant in the NCF-2 gene was detected. Resolution was achieved with antibiotic and antifungal therapy. The patient was lost to follow-up. |
| Castro CN | Case Series | Multiple Hospitals | 2 patients with novel NCKAP1L mutations | To describe a novel syndrome with HLH features caused by NCKAP1L variants | The study describes 2 unrelated patients of Middle Eastern origin presenting with symptoms of immunodeficiency, lymphoproliferation, and hyperinflammation. Clinical signs and laboratory tests were suggestive of HLH, so both patients received treatment according to the HLH04 protocol. No triggering factors were found. Functional and molecular tests were performed to understand the function of this protein. First report of NCKAP1L deficiency in humans. |
| Cesaro S | Case Report | Pediatric Onco-Hematology, University of Padova, Padova, Italy | 1 patient with 22Q11.2 Deletion Syndrome | To describe a case of HLH in a patient with 22q11.2 Deletion Syndrome | A 13-month-old child with a recent diagnosis of 22q11.2 deletion syndrome was admitted to the hospital for persistent fever and rash. Further evaluation showed signs consistent with secondary HLH. Treatment with HLH94 protocol was started, and the patient underwent HSCT. Unfortunately, he died at +22 years after HSCT due to severe VOD. |
| Celiksoy MH | Case Report | Department of Pediatric Immunology-Allergy, Ondokuz Mayis University, Samsun, Turkey | 1 patient with Ataxia Teleangiectasia | To describe a case of HLH in a patient with Ataxia Telangiectasia | A 3-year-old boy with a history of recurrent upper respiratory tract infections and otitis was admitted to the hospital for persistent fever and hepatosplenomegaly. On admission, he was found to have bilateral rales on chest auscultation. Further evaluation was consistent with HLH. He was treated with HLH04 protocol without achieving remission. He then underwent a haploidentical transplant from his mother, but unfortunately the patient died before grafting. Exome analysis confirmed the diagnosis of AT. |
| Cetinkaya PG | Case Series | Hacettepe University Faculty of Medicine, Ankara, Turkey | 28 patients with diagnosis of HLH underlying PID | To define mortality-related parameters in HLH secondary to PID | 28 patients diagnosed with HLH underlying IEI were included in the study. Twenty-seven patients met at least 5 of the 8 diagnostic criteria of HLH 2004. The patients were grouped according to the diagnosis of IEI: the first group consisted of patients with vesicular transport defects, such as GS2 and CHS, while the second group included patients with other IEI. The latter group included 21 patients. A low serum albumin level was the only variable associated with a 5.6-fold increase in mortality in patients with HLH secondary to IEI. |
| Chidambaram AC | Case Report | Department of Pediatrics, Jawaharlal Institute of Postgraduate Medical Education and Research (JIPMER), Puducherry, India. | 1 patient with ADA deficiency | To describe a case of HLH, DIC, AKI in a patient with ADA SCID | A 3-month-old girl with a history of bronchiolitis and watery diarrhea in the first two months of life was hospitalized for persistent fever and respiratory distress. She was subsequently diagnosed with CMV pneumonia with clinical and laboratory signs of HLH. She subsequently developed AKI and DIC. Her lymphocyte count was depressed, so a diagnosis of SCID was suspected. She received IVIG, dexamethasone, and ganciclovir for CMV infection. She died before starting treatment with etoposide for multi-organ failure. |
| Cui T | Case Report | Department of hematology, Beijing Friendship Hospital, Capital Medical University, Beijing, China | 1 patient with XIAP mutation | To describe a case of HLH in a patient with X linked lymphoproliferative syndrome | A 2-year-old boy was initially admitted to the hospital for suspected mononucleosis. After being treated with ganciclovir, steroids, and IVIG, the patient continued to manifest intermittent fever. He was then readmitted to the hospital where further evaluation showed signs consistent with HLH. The patient was treated with HLH04 and went into stable remission after 22 months of follow-up with no need for HSCT. The authors emphasize that precision therapy for secondary HLH in patients with XLP2 can control the flare without the need for HSCT quickly. |
| de la Varga-Martínez R | Case Report | Hospital Universitario Puerta del Mar, Cádiz, Spain | 1 patient with SH2D1A mutation | To describe a case of HLH in a patient with X linked lymphoproliferative syndrome | A 10-year-old boy from a family with no history of immunodeficiency was hospitalized for hepatitis, ascites, pleural effusion, hyponatremia, and seizures. He also presented with persistent fever and hepatosplenomegaly. Further evaluation was consistent with EBV-associated secondary HLH. He was treated according to the HLH04 protocol. Molecular analysis showed a novel variant of SH2D1A, confirming the diagnosis of XLP type 1. The outcome was good and now the patient is awaiting a new diagnosis. The outcome was good, and he is now awaiting HSCT. |
| Dvorak CC | Case Report | Lucile Packard Children’s Hospital, Stanford, CA | 1 patient with IL2RG deficiency | To describe a case of HLH in a patient with SCID and maternally engrafted T cells | The study describes a case of X-linked SCID in an infant who had hemophagocytosis associated with maternally incubated T cells in the bone marrow and in whom the classic lymphocytic phenotype of SCID was obscured by hemophagocytic syndrome. |
| Eng V | Case Report | Department of Allergy and Immunology, Kaiser Permanente Los Angeles Medical Center, Los Angeles, California. | 1 patient with STAT1 mutation | To describe a case of HLH in a patient with STAT1 gain of function mutation | A 6-year-old male had a significant history of congenital CMV, failure to thrive, pericardial effusion, and chronic pulmonary hypertension of unknown etiology, reactive airway disease, and enteropathy with dependence on total parenteral nutrition. He was evaluated for recurrent sinopulmonary infections and chronic mucocutaneous candidiasis. He was hospitalized for *CoNS* sepsis. Further evaluation suggested the presence of HLH. He was treated only with antibiotics. After 2 years he had a new flare-up of HLH during a pulmonary infection. For this second flare-up, he received the HLH protocol with resolution. Complete resolution was achieved with TCS. WES revealed the pathogenic variant STAT 1 with gain of function. |
| Escaron C | Case Series | Department of Bone Marrow Transplant, Great Ormond Street Hospital for Children, London, UK | 2 siblings with HLH underlying different mutations | To describe 2 cases of HLH in two siblings with different mutations | Two brothers who developed hemophagocytic lymphohistiocytosis due to distinct genetic abnormalities. Although their presentation was clinically similar, the study shows that a shared genetic diagnosis between siblings cannot always be assumed. Case 1, with definite FHL5, had seven of the eight HLH-2004 criteria. His brother presented at 4 years of age with XLP1 and EBV-triggered HLH and had six of the defining features of HLH. He died shortly after initiation of the HLH04 protocol due to general deterioration. |
| Gera A | Case Series | Division of Pediatric Hematology/Oncology, Department of Pediatrics, VMMC and Safdarjung Hospital, New Delhi, India | 9 patients with diagnosis of HLH and possible underlying PID | To describe clinical features of HLH in variable underlying diseases | The study is a retrospective review of children diagnosed with HLH. 3 of 9 had a genetic predisposition. AD Hyper IgE syndrome with HLH was described in an 18-month-old patient with a history of viral infections, fractures, hyper eosinophilia, and eczematous rash. No underlying cause was found. Most cases were treated according to the 2004 HLH protocol. |
| Gothe F | Case Report | Unclear setting | 1 patient with novel IFNAR1 mutation | To describe a case of HLH in a patient with IFNAR1 deficiency | A 15-month-old child presented with fever, rash, and tonsillitis. Further evaluation showed clinical and laboratory signs consistent with HLH. Bone marrow revealed no hemophagocytosis. No underlying cause was found. PCR on blood revealed a very low load for EBV. The patient was treated with antivirals, steroids, IVIG, cyclosporine A and anakinra with resolution. No mutations were found in familial HLH. Immediately after discharge, the patient had a new flare-up of inflammation associated with impaired renal function. He developed severe respiratory failure. At this point, genetic testing by WES was performed, identifying a nonsense mutation in IFNAR1 likely responsible for the hyperinflammation. |
| Greil J | Case Report | Department of Pediatric Oncology, Hematology and Immunology, University of Heidelberg, Germany | 1 patient with HMOX1 mutation | To describe a case severe hyperinflammation, microcytic anemia and defect in bilirubin synthesis in a patient with HMOX1 mutation | This study describes a patient carrying a G139V mutation in the active center of HO-1 that results in loss of normal HO-1 function and increased pathological peroxidase activity. Clinically, the mutation was associated with HLH at 18 months of age. Previously, in the first months of life, she had manifested persistent and profound microcytic anemia and splenomegaly. NK cell function in vitro showed reduced killing activity in unstimulated cells, but adequate killing following stimulation with IL-2. The HMOX1 mutation was then confirmed by molecular and functional assays. The authors emphasize the importance of HO-1 function in defense against oxidative stress and regulation of the macrophage-dependent inflammatory response. |
| Grunebaum E | Case Report | The Hospital for Sick Children and The University of Toronto, Canada | 1 patient with IL2RG deficiency | To describe a case of HLH in a patient with SCID | A 9-week-old male was admitted after 9 days of persistent fever and generalized seizures. Further evaluation was suggestive for HLH. Laboratory analysis showed profound lymphopenia. Western blot and flow cytometry demonstrated the absence of IL2R gamma chain. Molecular analysis confirmed the mutation of IL2RG. The patient was treated with etoposide and dexamethasone, but subsequently died of sepsis. |
| Halasa NB | Case Report | Vanderbilt Children’s Hospital, Nashville, TN, USA | 1 patient with SH2D1A mutation | To describe a case of HLH in a patient with X linked lymphoproliferative syndrome | An 18-year-old boy presented with symptoms, physical examination findings, and laboratory tests consistent with HLH caused by EBV. Molecular analysis detected a mutation in the SH2D1A gene. The boy was treated with etoposide, cyclosporine A, dexamethasone, and intrathecal methotrexate, according to the HLH-94 protocol, and had a transient remission of symptoms. He later developed EBV-associated necrotizing retinitis and sepsis due to Enterococcus, Klebsiella, and aspergillosis, which contributed to his death. |
| Han SP | Case Report | Taichung Veterans General Hospital, Taichung, Taiwan | 1 patient with X-linked agammaglobulinemia | To describe a case of recurrent HLH in a patient with XLA | A 7-year-old boy presented with a history of ecthyma gangrenosum and several episodes of pyogenic infections during childhood. At the age of 5 years, he developed multiple HLH for a total of 4 episodes of flares. The HLH episodes finally disappeared after receiving monthly Ig replacement therapy (400mg/kg) after the fourth HLH. In fact, profound hypogammaglobulinemia and depletion of circulating B cells was found at the second flare-up and one month after the first administration of IVIG. Molecular diagnosis confirmed the BTK mutation with a new mutation. Increased IgM was discovered incidentally after 6 cycles of monthly Ig replacement therapy. Multiple myeloma secreting IgM, Waldenström's macroglobulinemia, and lymphoma were excluded. |
| Harnisch E | Case Report | Sophia Children’s Hospital, Rotterdam, The Netherlands | 1 patient with ICF type 2 | To describe a case of HLH in a patient with ICF type 2 | An 11-year-old boy was hospitalized for persistent fever, hepatosplenomegaly, and generalized lymphadenopathy. History revealed recurrent respiratory infections, febrile seizures, neurodevelopmental delay, and a protracted CMV infection. EBV infection was diagnosed. Further evaluation was consistent with HLH. The patient was treated with dexamethasone, cyclosporine A and etoposide. He then underwent HSCT with 100% chimerism. The outcome was good. Molecular testing identified a novel ZBTB24 mutation. |
| Hügle B | Case Report | Department of Pediatrics, University of Leipzig, Leipzig, Germany | 1 patient with SH2D1A mutation | To describe a case of fulminant mononucleosis in a patient with X linked lymphoproliferative syndrome | A 4-year-old boy was hospitalized for severe infectious mononucleosis, later complicated by a virus-associated hemophagocytic syndrome. A biopsy on a lymph node identified histology compatible with Burkitt's lymphoma, but bone marrow aspirate ruled it out and found obvious signs of hemophagocytosis. In addition, a cerebellar tumor growth was found at the same time, which contributed to the patient's death. B-cell lymphoma was confirmed. The patient was treated according to the HLH94 protocol but died from severe intracranial hypertension. Molecular analysis on the parents confirmed the suspicion of XLP syndrome. The authors emphasize the uniqueness of simultaneous manifestation of XLP. |
| Higuchi T | Case Report | Department of Pediatrics, Kyoto University, Kyoto, Japan | 1 patient with XIAP mutation | To describe the use of IL18 as a diagnostic tool in a patient with HLH and XIAP mutation | A 1-month-old child was hospitalized for persistent fever and hepatosplenomegaly. After initial improvement with hydrocortisone, he relapsed after 12 days. Further evaluation was consistent with HLH. The elevated IL18 level led to suspicion of four conditions (XIAP, NLRC4, sJIA, CDC42). Target gene sequencing confirmed a known nonsense mutation in the XIAP gene. No additional pathogenic variants were found using a gene panel. The patient was treated according to the HLH protocol with resolution. He relapsed at 15 months. |
| Hoshino T | Case Report | Gunmaken Saiseikai Maebashi Hospital, Maebashi, Japan | 1 patient with SAP mutation | To describe a case of HLH in a patient with X linked lymphoproliferative syndrome | A 23-year-old man was admitted to the hospital for persistent fever. He had hypogammaglobulinemia and EBV-associated HLH. He was treated with steroids, chemotherapy, and immunoglobulin, but died 5 months after admission from liver and respiratory failure. Molecular analysis showed a single base substitution in the SH2D1A gene. In addition, the patient showed an alteration in type 1 T helper cells, as has been described in Sap KO mice. Th1/Th2 imbalance in humans, as well as in mice, could play an important role in the pathogenesis of XLP. |
| Honda K | Case Report | Hamanomachi Hospital, Fukuoka, Japan | 1 patient with SAP deletion | To describe a case of SAP deletion identified through FISH | A 15-month-old child was hospitalized for severe infectious mononucleosis. Further evaluation was consistent with EBV-related HLH. He was treated with dexamethasone, etoposide and then cyclosporine A, but unfortunately died from respiratory failure and bacterial infection associated with pancytopenia. The mother's brother died of viral disease at the age of 1 year. The patient was initially investigated by PCR analysis for the SH2D1A gene. DNA PCR for exons 1, 2, 3 and 4 of SAP revealed no amplification products, suggesting that the patient carried a large DNA deletion that included the SH2D1A gene. The deletion was confirmed by FISH. |
| Horneff G | Case Report | Centre for Pediatric Rheumatology, Department of Pediatrics, Sankt Augustin, Germany | 1 patient with TRAPS | To describe a case of recurrent MAS in a patient with TRAPS | An 11-year-old Turkish girl presented with fever, polyarthralgia, polyarthritis, hepatosplenomegaly, and rash. She received intensive treatment. Further evaluation was found to be compatible with MAS. Treatment according to HLH04 protocol with remission was then started. No functional abnormalities of NK cells were found. Thereafter, the patient had recurrent episodes of fever, arthralgias, and elevated CRP. At 14 years of age, she had a new flare-up of MAS. Treatment with anakinra was started with complete follow-up. Because of familiarity for fever episodes, genetic studies were conducted and a probable pathogenic point mutation in the TNFRSF1A gene was detected. |
| Imashuku S | Case Report | Kyoto City Institute of Health and Environmental Sciences, Mibu, Nakagyoku, Kyoto, Japan | 1 patient with WHIM syndrome | To describe a case of HLH and lymphoma in a patient with WHIM syndrome | WHIM syndrome was diagnosed in early childhood based on frequent infections, warts on hands and fingers, and myelokathexis. At 26 years of age, the patient developed high fever, multiple lymphadenopathies, and pleural effusion. Further evaluation was compatible with HLH from EBV and associated with clonal T-LPD. He was treated with prednisolone with resolution of HLH symptoms. Six weeks later he developed abdominal pain. He was then diagnosed with intestinal lymphoma. CHOP therapy proved ineffective. He died of intestinal perforation before HSCT. |
| Jain G | Case Report | Department of Pediatrics, Armed Forces Medical College, Pune, India | 1 patient with Chronic Granulomatous Disease | To describe a case of HLH in a patient with chronic granulomatous disease | A 6-year-old girl was admitted to the hospital with a history of prolonged fever, ear discharge, and recurrent pneumonia. On admission, necrotizing pneumonia was found in the apical lobe of the right lung and otitis media. The DHR test was suggestive of CGD. An antifungal was added, but the child continued to have high grade fever and developed HLH. No underlying cause was found. Antimicrobial therapy was changed to complete remission. No specific treatment for HLH was added. The outcome was good. |
| Jiang MY | Case Report | West China Second University Hospital of Sichuan University, Chengdu, China | 1 patient with BIRC4 mutation | To describe a case of HLH in a patient with BIRC4 mutation | A 5.8-year-old child, who experienced abdominal distension, fever, and pancytopenia, was hospitalized. Further evaluation suggested the presence of HLH. No underlying cause was found. He was treated with dexamethasone with complete resolution. Molecular analysis detected a two-nucleotide deletion in the BIRC4 gene, confirming XIAP deficiency. He then underwent HSCT. The child suffered from drug-associated enteritis while receiving the preparative regimen. GVHD, VOD, pulmonary hemorrhage and infection were not reported. The outcome was good. |
| Kashiwagi Y | Case Series | Department of Pediatrics, Tokyo Medical University, Tokyo, Japan | 4 patients with virus associated hemophagocytic syndrome | To describe cases of virus associated hemophagocytic syndrome in children and adolescence | This study identified 4 patients with virus-associated hemophagocytic syndrome. 2 of them had underlying conditions: combined immunodeficiency with predominant T-cell dysfunction and SLE. The patient with DIC was a 2-month-old child hospitalized for persistent fever and anorexia. Further evaluation suggested CMV hemophagocytic syndrome. The child was treated with high titer CMV gamma-globulin and pulse steroid therapy. Because CMV antigenemia was high, antiviral treatment was added. Before HSCT, he died of severe interstitial pneumonia. This study is also the first report of HLH due to parainfluenza and enterovirus in patients without known underlying conditions. The viruses were identified by molecular testing. |
| Klemann C | Case Report | Center for Chronic Immunodeficiency, Medical Center - University of Freiburg, Freiburg, Germany | 1 patient with ORAI1 mutation | To describe a case of HLH in a patient with ORAI1 mutation | A 3-month-old child was hospitalized for bronchiolitis and splenomegaly. A primary postnatal CMV infection was identified. Despite the administration of ganciclovir, the patient developed HLH. He was found to be a carrier of a heterozygous STX mutation, predicted to be benign. He was treated with dexamethasone with improvement. He later developed respiratory failure and was identified to have *P. jirovecii*, which required further diagnostic investigation. A novel ORAI1 mutation was identified. This is the first description of HLH in an ORAI1-deficient patient and illustrates that HLH syndrome can also occur in patients with severely defective T-cell activation. The authors point out that alteration of the luminal pupillary reflex, NK cell degranulation, and CD40L upregulation are helpful in diagnosing genetic disorders of CRAC channel function. |
| Kuijpers TW | Case Report | Emma Children’s Hospital, Amsterdam, The Netherlands. | 1 patient with TNFRSF6 mutation | To describe a case of HLH and CVID phenotype in a patient with FAS mutation | A 3-year-old boy was admitted to the hospital with a history of fever, hepatosplenomegaly, and lymphadenopathy after pneumonia. Further evaluation suggested the presence of HLH. HLH was treated with prednisone and IVIG. Once IVIG and prednisone were started, ferritin and sIL-2R levels did not completely normalize. He was investigated for IEI and found to have hypogammaglobulinemia and defective immune responses with absent memory B cells. All this was compatible with CVID. Subsequently, a mutation in TNFRSF6 was found. The authors point out that the CVID phenotype may be caused by FAS mutations. |
| Lam MT | Case Series | Multiple Hospitals | 4 patients with CDC42 mutation | To describe a new entity called NOCARH due to a novel CDC42 mutation | A novel hematologic/autoinflammatory condition (NOCARH syndrome) was described in four unrelated patients with similar features, including neonatal onset cytopenia with dyshematopoiesis, autoinflammation, rash and HLH. The patients shared the same de novo CDC42 mutation and alterations in the hematopoietic compartment, immune dysregulation, and inflammation. Emapalumab was critical to the survival of one patient, who underwent successful bone marrow transplantation. |
| Lekbua A | Case Report | Boston Children Hospital, Boston, MA, USA | 1 patient with BIRC4 mutation | To describe a case of VEO-IBD in a patient with BIRC4 mutation | The study describes a case of VEO-IBD secondary to a mutation in the BIRC4 gene in a 17-month-old child with severe growth failure, intractable diarrhea, and hepatosplenomegaly. Endoscopy and histology identified only mild duodenitis and ileitis but severe pancolitis with crypt abscesses and apoptosis of the epithelium. Minimal improvement of symptoms was achieved with total parenteral nutrition, steroids, and tacrolimus, while adalimumab therapy resulted in complete remission. After 6 months, the patient developed HLH and died of multisystem organ failure. |
| Le Voyer T | Case Series | Multiple Hospitals | 20 patients with STAT 1 AR LOF mutations | To describe STAT1 AR LOF clinical and functional characteristics through international survey | 20 unrelated patients selected through an international survey in 13 countries. 10 of them were previously described, while the others were described for the first time. 22 different mutations were found, and functional analysis confirmed complete or partial deficiency. 7 patients with AR STAT1 deficiency showed HLH triggered by live vaccines (MMR, VZV or BCG) or pathogens (HHV6, *C.parapsilosis*). For 1 patient, no triggering factor was found. 2 patients developed relapsed HLH. NK functional tests were normal in 75%. Two other patients had HLH: P11 developed fatal metapneumovirus-induced HLH after transplantation, and P30 died from BCG-induced hemophagocytic syndrome before further investigation or treatment was performed. |
| Liang J | Case Report | Department of Pediatrics and Pathology, Children’s Hospital of Pittsburgh of UPMC, Pennsylvania, USA | 1 patient with NLRC4 GOF mosaic | To describe a novel mutation of NLCR4 | A 28-week-old preterm infant with congenital anemia, ascites, and a heavily edematous placenta with fetal thrombotic vasculopathy developed hepatosplenomegaly and hyperinflammation suggestive of HLH in the early postnatal course and died at 2 months of age. The disease was presumed to have started in utero. Postmortem examination confirmed hepatosplenomegaly and hemophagocytosis at multiple sites. Whole-exome sequencing analysis demonstrated a novel NLRC4 de novo heterozygous mosaic mutation of NLRC4, which is believed to cause a dominant mutation, with gain of function and resulting constitutively active protein. |
| Liang J | Case Report | Department of Hematology, the First Affiliated Hospital of Nanjing Medical University, Jiangsu Province Hospital, Nanjing, China | 1 patient with SH2D1A mutation | To describe a case of HLH and NK cell leukemia in a patient with X linked lymphoproliferative syndrome | A 44-year-old woman was admitted to the hospital for fever, night sweats, weight loss, splenomegaly, and bilateral leg edema. Further evaluation was consistent with HLH. Concurrent NK-cell leukemia was diagnosed. The patient underwent chemotherapy, but unfortunately died after 5 months. Molecular analysis for HLH detected a SH2D1A mutation in the subject and a similar mutation in her brother. Western blotting analysis showed that the SAP protein was not expressed in the patient. |
| Loganathan A | Case Report | Department of Pediatric Hematology and Oncology and Pediatrics, Kanchi Kamakoti CHILDS Trust Hospital, Chennai, Tamil Nadu, India | 1 patient with SH2D1A mutation | To describe a case of HLH in a patient with X linked lymphoproliferative syndrome | An 11-month-old male infant, born to consanguineous parents, presented with intermittent fever and loose stools for 20 days. On admission, hepatosplenomegaly, bilinear cytopenia and elevation of liver enzymes were noted. Antibiotic therapy was started without effect. Further evaluation suggested the presence of HLH. No causative cause was found. The patient was treated according to the HLH protocol with dexamethasone, etoposide, and intravenous immunoglobulin, but died shortly thereafter from fulminant hepatic dysfunction and coagulopathy. Molecular analysis identified a mutation in SH2D1A. |
| Lougaris V | Case Report | Paediatrics Clinic and Institute for Molecular Medicine A.Nocivelli, University of Brescia, ASST-Spedali Civili of Brescia, Brescia, Italy | 1 patient with APDS | To describe a case of pediatric MAS/HLH underlying APDS | A 12-year-old boy, whose medical history showed no problems, began to complain of arthritis and arthralgias. After 4 months, he was hospitalized for fever, diffuse rash, and scaling. Further evaluation was consistent with MAS/HLH. He was treated with steroids, but later developed a new febrile flare-up. High-dose steroid treatment was repeated with a good clinical response, and he was added anakinra for 8 weeks, but then developed a new flare-up. Infectious etiologies were ruled out. PET scan was suggestive of lymphoma, but histology ruled it out. Immunologic analysis identified altered peripheral distribution of B lymphocytes. NGS sequencing revealed a novel mutation in the adapter-binding domain (ABD) linker region of p110δ. |
| Maignan M | Case Report | Department of Medicine, Hopital Sacre Cœur, University of Montreal, Montreal, QC, Canada | 1 patient with Chronic Granulomatous Disease | To describe a case of HLH in a patient with undiagnosed CGD | A 21-year-old man was hospitalized for *Burkholderia cepacia* complex pneumonia. Despite antibiotic treatment, the fever continued, and the patient's condition worsened. Anemia and thrombocytopenia developed along with hypofibrinogenemia. The patient died of multiple organ dysfunction 17 days after admission. Autopsy revealed hemophagocytosis, suggesting the diagnosis of HLH. DNA analysis showed a deletion in the p47phox gene. |
| Malkan UY | Case Report | Department of Hematology, Faculty of Medicine, Hacettepe University, Ankara, Turkey | 1 patient with CVID | To describe a case of CVID associated HL complicated with HLH | A 42-year-old male patient with CVID was investigated for back pain, splenomegaly, and diffuse lymphadenopathy. He was found to have Hodgkin's lymphoma (HL). He was given six cycles of chemotherapy, and complete remission was observed in the control CT. However, pancytopenia progressed, and he subsequently developed EBV-related HLH. He received an HSCT but died from transplant-related toxicity. |
| Marsh RA | Case Series | Division of Bone Marrow Transplantation and Immune Deficiency, Cincinnati Children's Hospital Medical Center, Burnet Ave., Cincinnati, OH, USA | 10 patients with BIRC4 mutations | To describe cases of HLH among patients with BIRC4 mutations | This is a retrospective study that identified 10 patients from 8 unrelated families with BIRC4 mutations followed from a single institution. Nine of the 10 patients developed HLH. Most patients presented in childhood, and recurrent HLH was common. Three patients died. Central nervous system involvement was frequent among these patients. |
| Martin A | Case Series | Vall d’Hebron Hospital, Barcelona, Spain and, Maria Pia Hospital, Porto, Portugal | 3 patients with Chronic Granulomatous Disease | To describe 3 cases of CGD with visceral Leishmania and HLH | Three of 20 cases of CGD with VL and IAHS are reported: 2 XL patients residing in Southern Spain and 1 AR patient in northeast of Portugal. Outcome was good for 2 patients while P1 died due to multiorgan failure despite appropriate treatment with IVIG and amphotericin B. |
| Marzollo A | Case Series | Multiple Hospitals | 3 patients with Chronic Granulomatous Disease | To describe neonatal manifestations of CGD | The authors report the cases of three infants of two different races. One CYBA-deficient infant presented with necrotizing pneumonia, which required prolonged antibiotic treatment and resulted in fibrotic lung changes. The second patient, first of two siblings, developed fatal sepsis from *Burkholderia multivorans* and died at 24 days of age. The younger brother had a diagnosis of CYBB deficiency and had MAS/HLH without any infection, which could be controlled with steroids. The second part of this study is a review of the literature. |
| Mischler M | Case Report | Divisions of Critical Care Medicine and Hematology and Oncology, C. S. Mott Children’s Hospital, University of Michigan, Ann Arbor, Michigan; | 1 patient with suspected X linked Lymphoproliferative Syndrome | To describe a case of HLH in a patient with X linked lymphoproliferative syndrome | A previously healthy 17-year-old boy was admitted to the ICU and was thought to have sepsis. Antimicrobial therapy was then started, but his condition progressively worsened. Further evaluation was consistent with hypertrophic HLH in the context of suspected X-linked lymphoproliferative disease. Functional testing showed the absence of SAP protein expression. Molecular analysis did not detect mutations in the SH2D1A gene. HLH04 protocol was started. Intrathecal therapy for CNS involvement was also initiated, with clinical improvement. A new therapeutic approach with anti-TNF therapy was initiated. This case illustrates HLH as a mimic of sepsis. |
| Ozturk C | Case Report | Department of Pediatrics, Izmir Tepecik Education and Research Hospital, Izmir, Turkey | 1 patient with X-linked agammaglobulinemia | To describe a case of HLH in a patient with XLA | A 5-year-old boy was hospitalized for severe pneumonia. Further evaluation suggested the presence of secondary HLH. No underlying cause was found. He was treated with intravenous antibiotics and then with intravenous immunoglobulin. Immediately after HLH, he was diagnosed with XLA. A mutation in the BTK gene was present. The outcome was good. |
| Pasic S | Case Report | Department of Pediatric Immunology, Mother and Child Health Institute; and Institute of Microbiology and Immunology, Belgrade, Serbia | 1 patient with suspected X linked Lymphoproliferative Syndrome | To describe a case of HLH in a patient with X linked lymphoproliferative syndrome | A 2.5-year-old boy was hospitalized for night sweats and rash, followed by spiking fever, lower extremity pain, and lymphadenopathy. In the following months, he experienced persistent fever without resolution with broad-spectrum antibiotics. Systemic JIA was suspected, and he was given oral prednisone. Despite treatment, the patient remained febrile and later developed hepatosplenomegaly and thrombocytopenia. Six months after the first symptoms, HHV8-related HLH was diagnosed. Cytofluorimetric testing did not identify SAP protein expression. Before being sent to another center for HSCT, he developed a lymphoproliferative reaction. He responded to HLH04 therapy. |
| Pasic S | Case Report | Department of Pediatric Immunology, Mother and Child Health Institute; and Institute of Microbiology and Immunology, Belgrade, Serbia | 1 patient with WAS | To describe a case of HLH in a patient with Wiskott Aldrich syndrome | A 4-month-old male patient with Wiskott-Aldrich syndrome was admitted for fever associated with bilinear cytopenia and splenomegaly. Analysis of a bone marrow sample revealed extensive hemophagocytosis. A diagnosis of EBV-HLH was made and immunotherapy with HLH-94 protocol was initiated. Partial remission of HLH was achieved, but the patient developed several septic episodes, so etoposide was discontinued. One year after onset, the patient died of a fungal infection. |
| Pachlopnik Schmid JM | Case Report | Division of Immunology/Hematology/BMT, University Children’s Hospital, Zurich, Switzerland; | 1 patient with NEMO mutation | To describe a case of HLH in a patient with hypomorphic NEMO mutation | A 2-month-old child manifested hemophagocytosis triggered by *K.pneumoniae* with transient deficiency of NK activity. He presented with IgM monoclonal gammopathy. The number of T cells in the blood had steadily increased, probably due to expansion of peripheral T cells. Further evaluation identified the NEMO mutation. Resolution of HLH was achieved with antibiotic therapy and intravenous immunoglobulin. The authors point out that patients with hypomorphic NEMO mutations and repeated infections may present with inflammatory dysregulation. |
| Parekh C | Case Series | Division of Pediatric Hematology Oncology, Children’s Hospital Los Angeles, Los Angeles, California; USA | 3 patients with Chronic Granulomatous Disease | To describe cases of HLH in 3 patients with CGD | This is a retrospective review of patients with CGD, treated between 1999 and 2008. Three of the 17 patients developed HLH. No underlying cause was found for one of them, although he had pneumonia. The diagnosis of CGD was made before HLH. No mutations are listed. All patients received IVIG and intravenous steroids to achieve resolution of HLH. The outcome was good. No specific genetic tests for HLH were performed. |
| Patiroglu T | Case Report | Department of Pediatric Immunology, Erciyes University School of Medicine, Kayseri, Turkey | 1 patient with IL2RG deficiency | To describe a case of HLH in a patient with SCID | A 3-month-old child was admitted to the emergency department for fever and vomiting. Oral thrush and hepatosplenomegaly were noted on admission. Immunophenotyping led to the suspicion of T-B+NK SCID. Molecular analysis confirmed this, finding a mutation in the IL2RG gene. Blood culture revealed the presence of Candida albicans. The patient was treated with IVIG and broad-spectrum antibiotics. Antifungal prophylaxis was initiated. After two weeks of progressive improvement, the patient was transferred to the ICU for fever, cough, dyspnea, jaundice, and elevated liver enzymes. Further evaluation was consistent with HLH. Pseudomonas was found in the culture of tracheal aspirate. Organ transplantation (HSCT) was planned, but the patient died before transplantation at the age of 4 months. |
| Prader S | Case Series | University Children’s Hospital Zurich, Zurich, Switzerland | 2 patients with GATA2 deficiency | To describe cases of HLH in 2 patients with GATA2 deficiency | 2 patients with GATA2 haploinsufficiency with severe VZV infection and HLH-like disease are described. Patient 1 is an 8-year-old girl admitted to the hospital for abdominal pain, rash, and fever. In the previous month, the patient had undergone medical examination for persistent warts on her limbs, and recurrent furuncles were noted on that occasion. The patient's condition gradually worsened, and further evaluation was consistent with VZV-induced HLH. The criteria were met. The patient was treated with steroids and gradually recovered from hepatitis, pneumonia, and HLH. Molecular analysis detected the GATA2 mutation. Patient 2, a carrier of the GATA2 mutation, did not show full HLH criteria, but he is also believed to have developed VZV-induced HLH. |
| Prader S | Case Report | Multiple Hospitals | 1 patient with SH2D1A mutation | To describe a case of HLH in a patient with X linked lymphoproliferative syndrome | A 6-year-old male patient meeting the diagnostic criteria of MIS-C was initially treated according to current consensus guidelines. The presence of hypofibrinogenemia, normal lymphocyte count and C-reactive protein, but elevated ferritin levels distinguished this patient from others with MIS-C. The clinical course following the initial presentation with ARDS was characterized by fatal liver failure in the setting of EBV-associated HLH, despite treatment with steroids, intravenous immunoglobulin, IL-1 receptor blockade, and rituximab. He died on the 8th day after admission. |
| Qiu KY | Case Report | Department of Pediatrics, Sun Yat-sen Memorial Hospital, Sun Yat-sen University, Guangzhou, P.R, China | 1 patient with CD40L mutation | To describe a case of Crohn Disease and EBV-HLH in a patient with XHIM | A 5-year-old male patient with abdominal pain, diarrhea, and fever that began 7 months earlier was admitted to the hospital. He had previously undergone enteroscopy suggestive of Chron's disease. He was initially treated with steroids and ASA. Upon admission, further testing suggested EBV HLH, while colonoscopy demonstrated chronic segmental inflammation. The patient was diagnosed with CD complicated by HLH. He received the HLH-2004 protocol and achieved partial remission, but soon relapsed. Eventually, the child's parents asked him to leave the hospital where he was to undergo HSCT. The outcome is unclear. Molecular analysis showed a mutation in the CD40L gene. |
| Razaghian A | Case Report | Department of Pediatrics, Division of Allergy and Clinical Immunology, Tehran University of Medical Sciences, Tehran, Iran | 1 patient with IFNGR1 deficiency | To describe a case of HLH in a patient with MSMD | A 2-month-old female patient was admitted to the hospital for fever, bilinear cytopenia, and hepatosplenomegaly. Further evaluation was suggestive for HLH. She had received the BCG vaccine at birth. This triggered HLH. She was treated according to HLH94 protocol but died of multi-organ failure at 4 months. A complete immunologic workup was performed to identify the underlying IEI. WES showed a homozygous mutation in IFNGR1. |
| Ren Y | Case Report | Myelodysplastic Syndromes Diagnosis and Therapy Center, Department of Hematology, The First Affiliated Hospital, Zhejiang, China | 1 patient with LRBA mutation | To describe a case of HLH in a patient with CVID | A 46-year-old woman with CVID caused by an LRBA mutation was hospitalized for persistent fever and fatigue and cytopenia for 20 days. Physical examination showed hepatosplenomegaly. Further evaluation was consistent with HLH. She was treated according to HLH04 protocol with improvement in spleen size but not cytopenia. She subsequently underwent haploidentical TCS from her older brother. The outcome was good. |
| Ricci S | Case Report | Department of Pediatric Immunology, Anna Meyer Children’s University Hospital, Florence, Italy | 1 patient with NEMO mutation | To describe a case of HLH in a patient with NEMO mutation | A 3-month-old baby was investigated for persistent diarrhea and failure to thrive. Umbilical separation was delayed. He presented clinical signs of anhidrotic ectodermal dysplasia. Radiologic evaluations were suggestive of osteopetrosis. Because he deteriorated rapidly, he was admitted to the intensive care unit where further evaluation was consistent with HLH. *P.jirovecii* and CMV DNA was found in the bronchoalveolar lavage by PCR. He was treated with systemic steroids and antibiotics/antivirals. An immunologic workup was performed at 4 months of age. A novel IKBKG missense mutation was identified. At 10 months, the patient suffered from E. coli bacteremia. At 13 months, he underwent haploidentical HSCT, but unfortunately died at +6 years of age from ARDS and sepsis due to P.aeruginosa. |
| Rossi-Semerano L | Case Report | Hopital de Bicetre, University of Paris Sud, Le Kremlin-Bicetre, France | 1 patient with FMF | To describe a case of MAS in a patient with Familiar Mediterranean Fever | A 4-year-old boy with a history of recurrent fever with arthritis and abdominal pain was hospitalized for elevated liver enzymes, pancytopenia, and coagulation abnormalities. Autoimmune hepatitis was initially suspected. Steroids and immunosuppressive therapy were then started. After transient improvement, she developed signs suggestive of MAS. Bone marrow aspirate confirmed the diagnosis. Cyclosporine was started, with rapid clinical and biological improvement. A few weeks later, the child developed acute episodes of fever associated with peritonitis, pleurisy, and systemic inflammation. The periodic fever was investigated, and it was found that the child carried a homozygous mutation in the MEFV gene. Steroids were reduced and colchicine was added with complete control. |
| Rudman Spergel A | Case Report | National Institutes of Health, Bethesda, Maryland, USA | 1 patient with ALPS | To describe a case of ALPS mimicking HLH features | This report describes a 6-year-old girl with HLH who was treated with chemotherapy before recognizing that her symptoms and laboratory values were consistent with a somatic FAS mutation leading to ALPS. The patient also carried a PRF1 gene variant of unknown significance. She was treated with chemotherapy for 22 months, then CT was discontinued. She later underwent splenectomy for discomfort due to splenomegaly, and histology raised the suspicion of ALPS. |
| Salzer E | Case Series | CeMM Research Center for Molecular Medicine, Austrian Academy of Sciences, Vienna, Austria | 8 patients with CD27 deficiency | To describe clinical phenotype of patients with CD27 deficiency | Eight pediatric patients from 3 independent pedigrees showed CD27 deficiency. Phenotypes ranged from asymptomatic memory B-cell deficiency to HLH from EBV, lymphoproliferative disorder and malignant lymphoma. The authors suggest that lack of functional CD27 predisposes to combined immunodeficiency associated with hemophagocytosis, lymphoproliferation, and development of potentially fatal EBV-induced lymphomas. Additional data are included in Seidel et al. 2012. |
| Schaballie H | Case Series | Department of Microbiology and Immunology, University Hospitals Leuven, Leuven, Belgium | 11 patients with Shwachman-Diamond syndrome | To describe mutations, clinical features, and the immunological profile of 11 patient with SD | 11 patients with SDS were retrospectively enrolled. This study confirms an understanding of the classic features of SDS, although the typical triad was present in only six of the nine patients studied. Four of the 11 patients were misdiagnosed with Jeune syndrome. Two patients presented with episodes of hypoglycemia. The immunophenotype was heterogeneous, although laboratory abnormalities were found in eight of ten patients. Three patients had life-threatening viral infection. In one patient, CMV caused HLH and severe cholestatic liver dysfunction at the age of 8 months. He was treated with ganciclovir. SDS was confirmed at age 13 years. |
| Scheffler-Mendoza SC | Case Report | Immunodeficiency Research Unit, National Institute of Pediatrics, Mexico City, Mexico | 1 patient with Chronic Granulomatous Disease | To describe a successful SCT in a patient with chronic granulomatous disease | An 18-month-old child with a history of severe infection was hospitalized for sepsis. Further evaluation suggested the presence of HLH. He was treated with dexamethasone and intravenous immunoglobulin. He subsequently underwent HSCT with complete resolution. Molecular analysis revealed a deletion involving both CYBB and XK genes, which encode the Kx antigen. The authors emphasize the importance of Kx antigen screening in patients with newly diagnosed CGD because the CYBB and XK genes are in the same region. Kx-positive transfusions can worsen anemia in patients with CGD-McPh. |
| Schmid I | Case Report | Kinderklinik and Kinderpoliklinik, Dr von Haunersches Kinderspital, University of Munich, Munich, Germany | 1 patient with T-B+NK+ SCID | To describe a case of HLH in a patient with SCID | A 5-month-old male presented with fever, hepatosplenomegaly, leukocytosis with atypical lymphoblasts, anemia and thrombocytopenia. T-, B+, NK+ SCID, EBV-triggered lymphoproliferative disease B and HLH were diagnosed. As her clinical situation rapidly deteriorated, a blood transplant was performed from her HLA-identical EBV-positive sister. Grafting occurred on day 6 with explosive proliferation of donor CD8(+) T cells. The patient died 3 days later from ARDS. Autopsy revealed complete incision by the donor and no signs of HLH or B lymphoproliferative disease. |
| Schultz KA | Case Series | Division of Hematology/Oncology and Blood and Marrow Transplantation, University of Minnesota, Minneapolis, Minnesota; USA | 2 patients with XLA | To describe cases of HLH in two brothers with underlying XLA | Two siblings developed HLH in the presence of underlying XLA and adenovirus infection. The youngest, a previously healthy 8-month-old male infant, was treated according to the HLH 04 protocol. Flow cytometry on the bone marrow detected the absence of B cells. Flow cytometry on the bone marrow detected the absence of B cells. The younger brother died of cardiopulmonary arrest. He died of cardiopulmonary arrest. The older brother was hospitalized a few days later for pneumonia, lethargy, and fever. He was also diagnosed with secondary HLH from Adenovirus. In his bone marrow, B cells were absent as in his brother. XLA was suspected and confirmed through cytofluorometric and genetic testing. |
| Seidel MG | Case Report | St Anna Children’s Hospital, Vienna, Austria, and Pediatric Hematology-Oncology, Medical University of Graz, Austria. | 1 patient with CD27 deficiency | To describe a case of HLH in a patient with CD27 deficiency | A 17-month-old girl, two months after undergoing infectious mononucleosis, developed clinical signs of HLH from EBV, pneumonia, systemic inflammatory response syndrome and LPD. She was treated with steroids. She showed moderate hypogammaglobulinemia, so replacement IVIG was started. After discontinuation of steroids, she developed life-threatening EBV-associated LPD. B cells were found to be devoid of CD27 and had a phenotype like CVID. For relapse of LPD from EBV, the child received Rituximab on two occasions. Additional data are included in Salzer et al. 2013. |
| Shadur B | Case Series | Hadassah University Medical Center, Department of Bone Marrow Transplantation and Cancer Immunotherapy, Jerusalem, Israel | 13 patients with SH2D1A or ITK mutation | To describe 13 cases of patients with SH2D1A or ITK mutations | Thirteen patients were discussed, including 10 with mutations in SH2D1A and three with mutations in the ITK gene. Patient 3 was diagnosed with XLP1 at 2 years of age based on family history and recurrent infections, fever, hypogammaglobulinemia and failure to thrive. He was well until age 32, when he developed fatal fulminant HLH unresponsive to HLH04 treatment protocol. Patient 13 was initially diagnosed with moderate to severe psychomotor retardation. At age 5, he was successfully treated for high-grade Hodgkin's lymphoma. He received chemotherapy, but six months later he represented EBV reactivation, recovering with Rituximab. At age 14, she developed EBV reactivation with extensive lymphoproliferation, and lymph node biopsy demonstrated Hodgkin lymphoma. He met criteria for HLH but, given his medical history, the family decided not to put him on intensive chemotherapy. He received rituximab, IVIg, and pulse steroids but died from multiorgan failure. Patient 11 died of fulminant HLH before the initiation of HLH04 protocol. |
| Shahin T | Case Series | Multiple Hospitals | 6 patients with IKZF2 mutations | To describe IKZF2 mutations in 6 patients with immune dysregulation | Germline mutations of IKZF2 have been described in six patients with systemic lupus erythematosus, immune thrombocytopenia or EBV HLH. The patients had hypogammaglobulinemia, decreased numbers of T-follicular helper and NK cells. RNA sequencing of a single PBMC cell from the patient carrying the R291X variant revealed upregulation of proinflammatory genes associated with T-cell receptor activation and T-cell depletion. Functional assays revealed the inability of Helios R291X to homodimerize and bind target DNA as dimers. Patient data are described in the supplements. |
| Shi B | Case Report | Department of PICU, Maternal and Child Health Hospital of Hubei Province, Women and Children’s Hospital of Hubei Province, Wuhan City, China | 1 patient with IL2RG deficiency | To describe a case of HLH in a patient with SCID | A 4-month-old child presented with fever and cough. Further evaluation was consistent with HLH. *Mycobacterium tuberculosis* complex was detected in peripheral blood by NGS metagenomics, and *M. bovis* was identified by polymerase chain reaction-reverse dot blot. The patient was then treated with Isoniazid, Rifampin and Pyrazinamide, but did not improve. However, the parents refused to accept further treatment, and the patient was discharged on the 12th day of hospitalization. To confirm the pathogenesis, a genetic analysis was performed. A mutation in the interleukin-2 receptor gamma subunit gene was detected. |
| Sheth J | Case Series | FRIGE’s Institute of Human Genetics, FRIGE House, Jodhpur Gam Road, Satellite, Ahmedabad, Gujarat, India | 2 patients with PRF1 or SH2D1A mutation | To describe 2 cases of HLH underlying PRF1 or SH2D1A mutation | P1, a 3-month-old baby born to second-degree consanguineous parents, was clinically suspected of HLH. A pathogenic variant in exon 2 of the PRF1 gene was detected. P2 was hospitalized and investigated for signs suggestive of HLH. Molecular study revealed a probable hemizygous pathogenic variant of the SH2D1A gene. The authors emphasize that patients presenting with EBV-associated HLH can be screened for XLP for early diagnosis and treatment implications. |
| Sieni E | Case Series | Meyer Children's Hospital, Florence, Italy | 11 patients with HLH underlying FHL or EBV related ID | To describe cases of HLH in patients with predisposing mutations | Eleven patients were diagnosed with FHL. They consisted of 9 males and 2 females. In 8 families, family history was not relevant at the time of diagnosis. 2 patients were affected by XLP1. Clinical, molecular, and functional data were described. The authors point out that FHL can present beyond pediatric age and up to the fifth decade. |
| Sirinavin S | Case Report | Departments of Pediatrics, Pathology and Radiology, Ramathibodi Hospital, Mahidol University, Bangkok, Thailand | 1 patient with Chronic Granulomatous Disease | To describe a case of HLH in adult patients with chronic granulomatous disease | A 17-month-old child was hospitalized for prolonged fever, hepatosplenomegaly, and a history of granulomatous lymphadenitis. During his hospitalization, he developed HLH. His clinical course mimicked tuberculosis, but the presence of *B.cepacia* was detected. He was treated with antibiotic therapy and intravenous immunoglobulin. After identification of *B.cepacia*, he was successfully treated with trimethoprim-sulfamethoxazole. The diagnosis of CGD was obtained by an NBT test. |
| Spinner MA | Case Report | Department of Medicine, Stanford University Medical Center, Stanford, California, USA | 1 patient with GATA2 deficiency | To describe a case of HLH and severe blastomycosis in a patient with GATA2 deficiency | An 18-year-old woman had a history of bilateral lower extremity lymphedema started at age 9 years and necrotizing Blastomyces pneumonia at age 12 years, when she was found to have mild neutropenia, lymphopenia, monocytopenia, and decreased NK cell function and number. NK deficiency was suspected. At 18 years of age, she developed severe HSV1-induced fatal HLH. She died despite appropriate antiviral and steroid therapy. Autopsy revealed hemophagocytosis in the bone marrow. Genetic testing performed posthumously confirmed a frameshift mutation of GATA2. |
| Squire JD | Case Series | Division of Allergy and Immunology, Department of Pediatrics, University of South Florida, St. Petersburg, FL, United States | 4 patients with Chronic Granulomatous Disease | To describe cases of HLH in 4 patients with chronic granulomatous disease | This is a series of pediatric X-linked CGD patients who developed HLH. In 2 patients, CGD was a known diagnosis before the development of HLH, while in the other 2 CGD was diagnosed as part of the evaluation for HLH through functional or genetic testing. 3 patients died and 1 underwent TCS. 2 of them had a family history of CGD. 1 had a history of granulomatous lymphadenopathy. One patient was previously described by Valentine et al. Pediatrics 2014. |
| Staines-Boone AT | Case Report | Immunology Department, UMAE 25 IMSS, Monterrey, Mexico | 1 patient with IFNGR1 deficiency | To describe a case of HLH in a patient with IFNGR1 deficiency | A 7-year-old boy who had multifocal osteomyelitis attributable to BCG since the age of 18 months was hospitalized for high fever, hepato-splenomegaly, lymphadenopathy, cytopenia, and hyperinflammation. He developed EBV-triggered HLH and died with multiorgan dysfunction. Because of his medical history, he was investigated for IEI and was found to have IFN-γ receptor 1 deficiency early in life, before the HLH episode. |
| Stepensky P | Case Series | Multiple Hospitals | 3 patients with ITK deficiency | To describe 3 patients with ITK deficiency | This study describes 3 cases from a single family who had EBV-positive B-cell proliferation diagnosed as Hodgkin's lymphoma. All three patients carried the same homozygous nonsense mutation of the ITK gene. One patient remained in stable remission, the second patient developed severe EBV HLH with multi-organ failure and died, and the third patient underwent successful allogeneic TCS. |
| Suzuki N | Case Series | Department of Pediatrics, Sapporo Medical University School of Medicine (N.S.), Sapporo, Japan | 20 newborn patients with HLH | To describe 20 cases of neonatal HLH | This study describes 20 newborn patients with HLH diagnosed within 4 weeks of life. The fever rate was low in preterm infants, and hypertriglyceridemia and neutropenia were uncommon. 6 patients had FHL. The overall survival rate of the 20 patients was 40%. There is one patient with SCID without genetic analysis. She developed HLH (criteria 5/8) on day 19 of life. She passed away despite various appropriate treatments at almost 3 months of age. |
| Szczawinska-Poplonyk A | Case Report | Department of Pediatric Pneumonology, Allergology and Clinical Immunology, Poznan University of Medical Sciences, Poznan, Poland | 1 patient with CDC42 mutation | To describe a novel mutation of CDC42 | An 11-year-old child with syndromic features, immunodeficiency, neurodevelopmental delay, and autoinflammation developed HLH and malignant lymphoproliferation. A novel mutation was found in the CDC42 gene by whole-exome sequencing. The patient was treated with pulsed MDP, etoposide, cyclosporine, antibiotics, antivirals, and antifungals. The outcome was good. |
| Tesi B | Case Series | Multiple Hospitals | 2 patients with IFNGR mutations | To describe 2 cases of HLH in patients with underlying IFNRGR mutations | The authors describe 2 unrelated cases of fatal IFN-g receptor deficiency with mycobacterial infections, initially diagnosed as HLH. Patient A was treated with steroids, cyclosporine and ATG with an initial remission, while patient B was treated with steroids, etoposide and ATG. Both patients died. Molecular analysis revealed a possible homozygous deletion of exon 2 of IFNGR2. By evaluation of IFNGR2 transcripts, the patient expressed only a truncated IFNGR2 transcript lacking exon 2. A missense variant of IFNGR1 was identified in patient B. |
| Triebwasser MP | Case Report | Division of Oncology, Children’s Hospital of Philadelphia, Philadelphia, Pennsylvania, USA | 1 patient with IKZF2 mutation | To describe the use of Emapalumab and Ruxolitinib in a patient with HLH underlying IKZF2 mutation | A 26-year-old man with CA-EBV-associated HLH, CNS involvement, refractory to HLH-2004 treatment and anakinra. He achieved remission with the new combination of Emapalumab and Ruxolitinib. He achieved complete disease remission and successfully underwent TCS with 100% donor chimerism. This is the first report on the combination of these two drugs. |
| Tucci F | Case Report | IRCCS San Raffaele Scientific Institute, Milan, Italy | 1 patient with ADA SCID | To describe the use of Emapalumab in a patient with ADA SCID | A 4-year-old girl with ADA-SCID complicated by disseminated TB underwent gene therapy. After failure of gene corrected cell engraftment, the girl received two HLA-haploidentical T-cell depleted HSCTs from her father, both of which failed due to GFs related to multiple concomitant infections and secondary HLH. Emapalumab administration controlled HLH and prevented GF after a third haploidentical HSCT from the mother. All infections improved with antimicrobial drugs and disseminated TB did not reactivate. The authors emphasize the use of Emapalumab for the treatment of HLH and prevention of GF in patients undergoing haplo-HSCT even with multiple infections, including TB. |
| Uslu N | Case Report | Hacettepe University, Faculty of Medicine, Department of Pediatrics, Section of Hematology, Ankara, Turkey | 1 patient with FMF | To describe a case of HLH in a patient with FMF | This case describes an 11-year-old pediatric patient with Crohn's disease on immunosuppressive therapy and FMF who developed fatal HLH. The patient was treated with antibiotics, steroids, cyclosporine, and intravenous immunoglobulin, but died at +41 days after admission due to progressive clinical deterioration, pancytopenia, and severe intestinal bleeding. The authors emphasize the importance of evaluating patients with inflammatory bowel disease receiving immunosuppressive therapy and presenting with unexplained fever, cytopenia, progression of organomegaly, and biochemical changes to investigate HLH for diagnosis and treatment. |
| Valentine G | Case Report | Section of Pediatric Critical Care Medicine, Department of Pediatrics, Baylor College of Medicine, Houston, Texas | 1 patient with Chronic Granulomatous Disease | To describe a case of HLH in a patient with chronic granulomatous disease | An 8-week-old Hispanic male was admitted to the hospital for FUO. He was diagnosed with HLH. No underlying cause was found. Immunosuppressive therapy was started, resulting in disseminated candida septic shock and sepsis-induced multisystem organ failure. Further evaluation finally established the diagnosis of CGD. The patient was treated with granulocyte CSF, immunoglobulin infusions and antifungals with resolution of the hyperactive inflammatory state. |
| van Montfrans JM | Case Report | Department of Pediatric Immunology, Wilhelmina Children’s Hospital/ University Medical Centre Utrecht, Utrecht, The Netherlands | 1 patient with Chronic Granulomatous Disease | To describe a case of HLH in a patient with chronic granulomatous disease | A 3.5-year-old male patient was diagnosed with HLH, which appeared to have developed during a multiorgan polymicrobial infection caused by *S.maltophilia* and *B.cepacia* due to an underlying diagnosis of X-CGD. He was treated according to the HLH04 protocol. He was found to carry a heterozygous mutation in the PRF1 gene. The authors speculate that the latter alteration serves as a genetic risk factor for HLH. Within 4 days of his HLH diagnosis, he developed severe hypoxic-ischemic brain injury with diffuse edema and brainstem herniation due to his coagulopathy. Unfortunately, he died. |
| Vavassori S | Case Series | Multiple Hospitals | 15 patients with ZNFX1 deficiency | To describe the clinical and molecular features of biallelic ZNFX1 deficiency | Fifteen patients from 8 unrelated families with history of severe infections and virus-triggered inflammatory episodes with HLH and HLH-like disease, early-onset seizures, renal and pulmonary disease were studied. WES was performed in 13 patients. The authors analyzed the transcriptome, post-transcriptional regulation of interferon-stimulated genes, and susceptibility to viral infections in primary cells from patients and controls stimulated with synthetic double-stranded nucleic acids. Homozygous and heterozygous compound deleterious variants of ZNFX1 were identified in all 13 patients. Six patients developed episodes of HLH; a trigger was found for 5 patients. The outcome was negative for 5 of 6 patients. |
| Vieth S | Case Report | Department of Pediatrics, UKSH, Campus Lübeck, Germany | 1 patient with BIRC4 mutation | To describe clinical phenotype and functional analysis of a XIAP/BIRC4 mutation | A 5-year-old child was hospitalized for persistent fever, night sweats, and lymphadenopathy. Further evaluation was consistent with HLH. His medical history was characterized by 2 episodes of respiratory infections and thrombocytopenia, elevated ferritin and sIL2 levels. He had previously been studied for EBV-related disorders without results. He had a family history of death at a young age due to infection. He was treated with steroids and empirical antibiotics. Later molecular testing was repeated, which identified a novel BIRC4 mutation. The outcome was good. |
| Vignesh P | Case Report | Allergy Immunology Unit, Department of Pediatrics, Advanced Pediatrics Centre, Postgraduate Institute of Medical Education and Research, Chandigarh, India | 6 patients with SCID | To describe clinical and laboratory features of SCID cases who developed HLH. | Six out of 94 cases with SCID over a 20-year period developed HLH-like manifestations. The male-to-female ratio was 5:1. A history of unexpected death was found in 4 cases. Molecular defects in IL2RG were found in 5 of 6 cases. Documented infections included BCGitis, bloodstream infections (*Staphylococcal aureus, Klebsiella pneumonia* and *Pseudomonas aeruginosa*), pneumonia. All patients died despite appropriate treatment. |
| Voeten M | Case Report | University of Antwerp, Antwerp University Hospital, Department of Pediatrics, Antwerp, Belgium | 1 patient with SH2D1A mutation | To describe CNS involvement due to HLH in a patient with X linked lymphoproliferative syndrome | A 3-year-old boy with encephalitis, elevation of cerebrospinal fluid protein endpoint, and diffuse brain MRI abnormalities was studied. He was initially treated with steroid therapy, which did not yield results. He then developed signs and symptoms suggestive of HLH. The diagnosis was not established until 6 weeks after the initial presentation. The boy recovered after therapy for HLH with the persistence of mild cognitive defects. Molecular analysis revealed SH2D1A mutation as the cause of HLH. The outcome was good after HSCT. This is the first report of an extremely high level of protein in CSF. |
| Wegehaupt O | Case Report | Center for Pediatrics and Adolescent Medicine, Medical Center, Faculty of Medicine, University of Freiburg, Freiburg, Germany | 1 patient with TIM3 deficiency | To describe a case of TIM3 deficiency with recurrent subcutaneous panniculitis-like T-cell lymphoma and HLH | A 17-year-old male patient presented with panniculitis-like T-cell lymphoma involving mesenteric adipose tissue associated with HLH. He was initially treated with etoposide with clinical resolution. Eight years later, he developed clonally unrelated SPTCL and underwent HSCT. Retrospectively, he was found to be carrying germline mutations in HAVCR2 associated with low TIM-3 expression. The authors point out that localization in mesenteric adipose tissue of SPTCL may be the manifestation of TIM-3 deficiency, that this condition predisposes to recurrent lymphoma, and that flow cytometry is a possible screening tool. |
| Wei A | Case Report | Discipline of Pediatrics, Beijing Children’s Hospital Affiliated to Capital Medical University, Beijing, China | 1 patient with Chronic Granulomatous Disease | To describe a case of HLH in a patient with chronic granulomatous disease | A 3-year-old boy was hospitalized for fever, hepatosplenomegaly, and pancytopenia. Further evaluation was suggestive for HLH. Blood and bone marrow culture confirmed sepsis due to Salmonella Typhimurium. Treatment was based on antimicrobials and methylprednisolone to control HLH. Etoposide was avoided because of sepsis. After treatment, clinical symptoms and laboratory findings improved. Genetic analysis showed a novel hemizygous mutation in the CYBB gene. |
| White S | Case Report | OSF Healthcare Children’s Hospital of Illinois, Peoria, Illinois, USA | 1 patient with STAT3 mutation | To describe a case of HLH in a patient with Hyper-IgE syndrome | A 7-year-old boy, previously diagnosed with HIES due to an autosomal dominant STAT3 mutation, was transferred to the ICU for septic shock. Further evaluation was consistent with EBV-induced HLH. He was treated according to the HLH94 protocol. |
| Yang X | Case Series | Department of Pediatrics, Graduate School of Medicine and Pharmaceutical Sciences, University of Toyama, Toyama, Japan | 3 patients with XIAP mutation | To describe a case of incomplete HLH in a female with XIAP mutation | Three siblings, two males and one female, with pancytopenia have been described. They are carriers of a novel XIAP mutation. All three patients showed deficient expression of XIAP protein. In the female patient, the paternally derived X chromosome was non-randomly and exclusively inactivated in peripheral blood and hair root cells. This is the first report of a female patient with incomplete HLH resulting from a heterozygous mutation of XIAP in association with non-random XCI. Her siblings developed EBV-induced secondary HLH. They were treated with prednisolone with resolution. No outcome data are available. |
| Yang X | Case Series | Department of Pediatrics, Graduate School of Medicine and Pharmaceutical Science, University of Toyama, Toyama, Japan | 2 patients with SH2D1A/XIAP mutations | To characterize EBV infected cells of 2 patients with HLH and XLP1 and XLP2 | EBV-infected cells in two patients with XLP-1 and XLP-2 who had EBV-HLH were evaluated by EBER-1 in situ hybridization or quantitative PCR methods. Both patients with XLP showed that the dominant population of EBV-infected cells were CD19+B cells, while EBV-infected CD8+T cells were in very low numbers. The authors emphasize that B-cell-directed therapy, such as rituximab, may be a viable option in the treatment of EBV-HLH in patients with XLP. |
| Yao J | Case Series | Hematology Center, National Center for Children`s Health, Beijing Children`s Hospital, Capital Medical University, Beijing, China | 5 patients with LRBA mutation | To describe 5 cases of patient with LRBA mutation | Five patients with heterozygous variations of LRBA were described. Hypogammaglobulinemia was recorded in four patients, and the percentage of T reg decreased in two patients. Only one patient showed an increase in double-negative T cells. Lymphoproliferative manifestations were observed in three patients. All patients had cytopenia with different clinical manifestations. None of the parents were asymptomatic. P5 also had recurrent infections and an autoimmune endocrinopathy. P3 showed HLH and received first-line chemotherapy. |
| Zhang R | Case Series | Hematology and Oncology Laboratory, Beijing Pediatric Research Institute, Beijing Children’s Hospital, Capital Medical University, National Center for Children’s Health, Beijing, China | 4 patient with PIK3CD mutation | To describe clinical and laboratory features of PIK3CD mutation in 4 patients | Four unrelated pediatric patients carrying PIK3CD mutations were enrolled in the study. WES identified a common pathogenic GOF mutation of P110delta for 3 of them. The fourth patient inherited a novel mutation in the C2 domain of p110delta from his father. His pathogenesis is uncertain because his father is asymptomatic. He also carries a heterozygous RAB27A mutation inherited from his mother, who is asymptomatic. This fourth patient developed EBV-related T lymphoproliferative disease complicated by HLH. He was treated according to HLH protocol 04. He then received an L-dep regimen but relapsed. He underwent HSCT with complete resolution. |
| Zheng F | Case Report | Department of Pediatrics, Union Hospital, Tongji Medical College, Huazhong University of Science and Technology, Wuhan, China | 8 patients with ITK deficiency | To describe cases of HLH in patients with underlying predisposing mutations | This is a retrospective study of 8 cases of severe HLH from a pediatric intensive care unit (PICU) over a 1-year period. EBV was the most common etiology. All patients underwent genetic analysis, which showed that four patients with EBV infection had a homozygous mutation in the ITK gene, with reduced survival rates. The group with ITK+ mutation had higher percentages of CD3+ CD8+ T cells than the group with ITK mutation, while they had similar levels of CD3+ CD4+ T cells. The ITK+ mutation group had lower percentages of CD3 cells. CD19+ B cells and CD16+ CD56+ NK cells compared with the ITK mutation group. EBV-infected patients with c.985+75G>A mutation in ITK had lower survival rates than the group with mutation c.985+75G>A in ITK. EBV-infected patients with c.985+75G>A mutation in ITK had lower survival rates than the group with ITK mutation, which could be related to cellular immune dysfunction. 3 patients with ITK mutation died. |
| Zhou S | Case Report | Key Laboratory of Birth defects Prevention, National Health and Family Planning Commission, Zhengzhou, China | 1 patient with SH2D1A mutation | To characterize a novel mutation of SH2D1A | A 3-year-old child was hospitalized for persistent fever. Further evaluation was suggestive for HLH. Multiple genetic tests revealed a deletion in the SH2D1A gene. No information on outcome or treatment is reported. |
| Zhou Z | Case Report | Department of Immunology, Laboratory Medical Immunology, Erasmus University Medical Centre, Rotterdam, The Netherlands | 1 patient with PIK3CD mutation | To describe a case of HLH in a patient with APDS | A 19-year-old man was evaluated for chronic active EBV disease. Four months before presentation, he developed fatigue, fever, night sweats, hepatosplenomegaly, anemia, hepatitis, weight loss, and lymphadenopathy. During work up for PID, he was found to carry a heterozygous mutation in the PIK3CD gene, which is associated with APDS. He was treated with rituximab. After 3 months, he was hospitalized for HLH. He was treated with high-dose steroids, sirolimus, and intravenous immunoglobulin. He died 1 month later from heart failure and refractory pulmonary edema. |
